# Impact of exercise training in a hypobaric/normobaric hypoxic environment on body composition and glycolipid metabolism in individuals with overweight or obesity: a systematic review and meta-analysis

**DOI:** 10.3389/fphys.2025.1571730

**Published:** 2025-03-10

**Authors:** Peng Liu, Hao Chen, Xin Jiang, Jorge Diaz-Cidoncha Garcia

**Affiliations:** ^1^ College of Physical Education, Dalian University, Dalian, China; ^2^ Department of Physical Education, Dalian University of Finance and Economics, Dalian, China; ^3^ Graduate School, Beijing Sport University, Beijing, China; ^4^ Fédération Internationale de Football Association (FIFA), Zurich, Switzerland

**Keywords:** hypoxia training, obesity, body composition, metabolism, meta analysis

## Abstract

**Objective:**

This study aims to assess the impact of hypoxia training on body composition and glycolipid metabolism in excess body weight or living with obese people through meta-analysis.

**Methods:**

Randomized controlled trials investigating the effects of hypoxia training on body composition, glucose, and lipid metabolism in excess body weight or living with obese people were systematically searched from databases including CNKI, PubMed, and Web of Science. The meta-analysis was performed by using Stata 18 and RevMan 5.4 analytic tools. The risk of bias was assessed using the Cochrane evaluation tool, and the level of certainty of evidence was determined by the GRADE framework. Between-study heterogeneity was examined using the *I* ^2^ test, and the publication bias was evaluated via the Egger test or funnel plot.

**Results:**

A total of 32 RCTs with 1,011 participants were included. A meta-analysis of 25 RCTs was performed (499 men and 480 women, Age: 40.25 ± 15.69, BMI: 30.96 ± 3.65). In terms of body composition, the outcome indexes of body fat ratio (MD is −1.16, 95% CI -1.76 to −0.56, *P* = 0.00) in the hypoxia group were better than the normal oxygen group. There was no significant difference in body mass and BMI between the hypoxia group and the normal-oxygen group (*P >* 0.05). In terms of lipid and glucose metabolism, no significant changes were found between the hypoxia group and the normoxia group (*P >* 0.05). Subgroup analysis showed that training in hypoxic environment at altitude 2001–2,500 m could effectively improve body mass, TG and LDL-C (*P* < 0.05). The effective program to reduce body mass is to carry out moderate intensity training of 45–60 min for ≤8 weeks, ≥4 times a week (*P* < 0.05).

**Conclusion:**

Hypoxic training is essential for reducing body fat ratio in excess body weight or obese people. It is recommended to carry out 45–60 min of moderate-intensity aerobic exercise for ≤8 weeks, ≥4 times a week, in a hypoxia environment of 2,001–2,500 m to lose body mass. The effects of hypoxia training and normoxia training on lipid and glucose metabolism in excess body weight or obese people are the same.

**Systematic Review Registration:**

https://www.crd.york.ac.uk/PROSPERO/view/CRD42024628550

## 1 Introduction

In the clinical field, Excess adiposity has gradually become a global public health issue and a leading cause of death in most countries’ populations (Chen et al., 2022). The World Health Organization (WHO) defines excess body weight and excess adiposity by body mass index (BMI), individuals with a BMI exceeding 30 kg/m^2^ are typically classified as living with obesity, while those with a BMI ranging from 25 to 30 kg/m^2^ are considered excess body weight (Badimon et al., 2017). In recent years, alongside economic development and improved living standards, the prevalence of excess body weight and excess adiposity has continued to rise (Tee et al., 2023; Collaboration, 2016). The World Health Organization forecasts that by 2030, nearly 60% of the global population will be excess body weight or with obesity (Dragano et al., 2020). Excess body weight and excess adiposity can impair their exercise capacity and, coupled with excessive caloric intake, elevate the risk of cardiovascular ailments like diabetes mellitus and hypercholesterolemia (Chacaroun et al., 2020; Chen et al., 2022; Wang and Sun, 2019; Wang et al., 2012), consequently augmenting healthcare expenses and societal and economic burdens (Wang et al., 2021; Wang et al., 2011). Additionally, they may encounter discrimination from society, potentially impacting their mental wellbeing and reducing their quality of life (Heymsfield and Wadden, 2017; Han, 2020; Jin et al., 2023). Therefore, implementing appropriate interventions to manage people’s weight and enhance their glucose and lipid metabolism is imperative. Exercise for weight loss is globally important (Exercise for weight loss was no. One in China, Brazil, Mexico, and Spain and no. Four in Europe) (Kercher et al., 2021). Exercise aids in preserving lean body mass through weight loss efforts and helps consumers maintain long-term weight loss (Newsome et al., 2024). For excess body weight or obese individuals, although aerobic exercise can bring about effective weight loss, it usually takes a relatively long time; in contrast, interval training is an efficient and time - saving exercise option (Batrakoulis et al., 2021), and combined training has the most positive influence on cardiometabolic health indicators (Batrakoulis et al., 2022).

A hypoxia environment refers to an environment with reduced oxygen partial pressure compared to the normal sea-level oxygen environment, encompassing both natural high-altitude hypoxia environments and artificial hypoxia environments (Wang and Sun, 2019). Hypoxic exercise training is a method of exercise and fitness in a naturally occurring or artificially simulated plateau where the body is below normal oxygen conditions (Weng et al., 2006). Individuals accustomed to living at sea level may experience altitude sickness when first exposed to high altitudes, making altitude training impractical due to its associated challenges in distance and duration. Consequently, in recent years, artificial simulation of hypoxia environments, rather than natural high-altitude conditions, has been demonstrated to enhance athletes’ aerobic and anaerobic exercise capacities (Faulhaber et al., 2023; Feng et al., 2023; Mujika et al., 2019)and performance and is emerging as a potent non-pharmacological intervention against numerous diseases (Burtscher et al., 2023). This artificially simulated hypoxic environment is also progressively utilized to enhance the body composition and lipid metabolism of living with obese individuals. Research indicates that prolonged residence in high-altitude, hypoxia environments results in weight loss (Xie et al., 2017).

When the load is regulated by heart rate, hypoxic environments typically induce a slight increase in heart rate, potentially alleviating the body’s burden compared to normal oxygen conditions (Yang et al., 2014). Exercising in a hypoxic environment also creates greater metabolic strain and increases overall fatigue (Ruggiero et al., 2022). Furthermore, many training modalities in hypoxic environments involve stationary bicycles, which can mitigate the risk of bone, knee, and ankle injuries in excess body weight or living with obese individuals (Yan, 2020). At the same time, it is feasible, safe, and effective for excess body weight/with excess adiposity individuals (Batrakoulis and Fatouros, 2022). Previous studies have shown that training in the normobaric hypoxic environment does not produce better benefits (Chen et al., 2022). There are also no studies exploring the best hypoxic training prescriptions for people who are excess body weight or obese. Hence, this study aims to assess the impact of hypobaric/normobaric hypoxic training on enhancing body composition and glucose and lipid metabolism in excess body weight or living with obese people via meta-analysis, aiming to ascertain its efficacy compared to conventional oxygen training and explore the best hypoxic training program for excess body weight or obese people.

## 2 Materials and methods

This paper follows the Systematic Review and Meta-Analysis Project (PRISMA) guidelines (Page et al., 2021). The PROSPERO registration number is CRD42024628550.

### 2.1 Inclusion and exclusion criteria

Inclusion criteria: Inclusion criteria followed the PICOS principles. (1) The participants were overweight or obese, and had no physical restrictions or health conditions that would preclude evaluation and exercise intervention; (2) Clinical randomized controlled trials; (3) In a hypobaric/normobaric hypoxic environment, oxygen concentration ≤17.4% or altitude ≥1,500 m; (4) The experimental group underwent hypoxia training hypoxic environme, while the control group received normal oxygen training; (5) Outcome: body mass, body fat ratio, body mass index, total cholesterol, triglycerides, low-density lipoprotein cholesterol, high-density lipoprotein cholesterol, fasting blood glucose, fasting blood insulin, and homeostatic assessment of insulin resistance, there is at least one outcome indicator in the literature.

Exclusion criteria: (1) Review Literature; (2) Case report studies; (3) Animal experimental literature; (4) Non-randomized controlled trials.

### 2.2 Literature search

A literature search was performed on databases including China National Knowledge Infrastructure (CNKI), Pub Med, and Web of Science, covering the period from the inception of each database to 11 January 2024. The last literature search was conducted on 1 December 2024. We use the exact Boolean operator (AND, OR) to concatenate search terms. The following combination of terms was used: “hypoxia training” or “hypoxia exercise” or “Intermittent hypoxia” or “altitude training”. The Boolean operator “AND” was used to combine these descriptors with “overweight” or “obesity” or “obese”. References included in the study and meta-analysis were also manually checked to avoid possible missing studies.

### 2.3 Literature screening and data extraction

The literature was retrieved from the databases using the established search strategy. Duplicate literature was removed using End Note X9 software, and irrelevant articles were excluded based on the examination of their titles and abstracts. Subsequently, articles that did not meet the inclusion and exclusion criteria were excluded.

Data extraction included retrieving information such as the first author’s name, publication year, sex distribution, sample size, age distribution, body mass index, intervention duration, intervention frequency, altitude, exercise modality, training duration, and outcome measures.

### 2.4 Risk of bias assessment for included studies

The Cochrane Collaboration RCT bias evaluation tool in Revman 5.4 software was employed to assess: (1) Random sequence generation; (2) Allocation concealment; (3) Blinding of participants and personnel; (4) Blinding of outcome assessment; (5) Incomplete outcome data; (6) Selective reporting; (7) Other bias. (This assessment was conducted independently by two researchers, with discrepancies resolved through discussion with a third researcher.)

### 2.5 Certainty of evidence

The Grading of Recommendations Assessment, Developing and Evaluation (GRADE) method was employed to assess the quality of evidence. GRADE assesses the certainty of evidence to fall into the categories of very low, low, moderate, or high.

### 2.6 Statistical analysis

The outcome indicators extracted from the included literature were analyzed using Stata 18 software. Since the outcome indicators in the included studies are continuous variables, the unit of body composition was the same, so mean difference (MD) was used as the effect size. The units of lipid and sugar metabolism are different, and the effect sizes (Hedges’s d) were expressed as standardized mean difference (SMD) and 95% confidence intervals (CI). Heterogeneity among outcome indicators was assessed using *I* ^2^ statistics. When *I* ^2^ < 50%, indicating small heterogeneity, the fixed-effect model was employed for analysis. Conversely, if *I* ^2^ suggested significant heterogeneity, exceeding 50%, the random-effects model was utilized. The source of atypia was identified by sensitivity analysis. The subgroup analysis method was used to explore the best hypoxic fat reduction training prescription. Publication bias was assessed by funnel plot or Egger’s regression test.

## 3 Results

### 3.1 Literature search process and screening results

**FIGURE 1:**
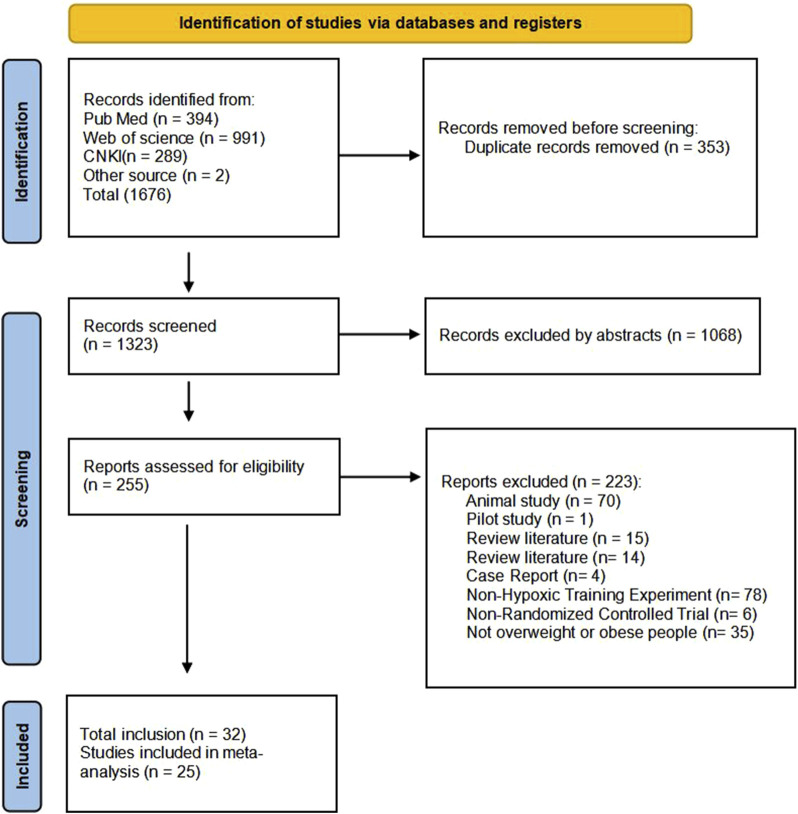
Flow diagram of the process of article selection.

A total of 1,676 articles were retrieved from databases (Specific literature search strategies are shown in Supplementary Table 1). After excluding 353 replicated studies, the texts of the remaining articles were reviewed. Ultimately, 32 studies met the inclusion criteria and were included in the analysis. The flow chart illustrating the literature screening process is presented in Figure 1.

### 3.2 Basic information and risk of bias assessment results of included literature

**FIGURE 2:**
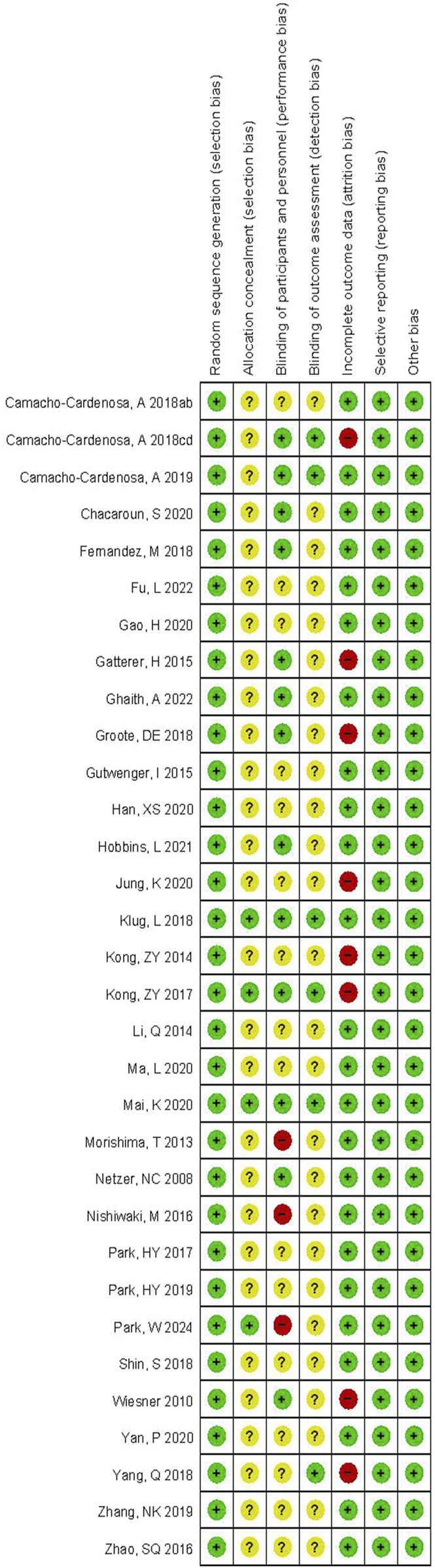
The risk assessment of bias.

The basic information of the literature included in this study is presented in Supplementary Table 2. Of the 32 randomized controlled studies included (a meta-analysis of 25 studies (Park et al., 2024; Ghaith et al., 2022; Fu and Li, 2022; Hobbins et al., 2021; Ma, 2020; Jung et al., 2020; Gao et al., 2020; Chacaroun et al., 2020; Park et al., 2019; Zhang, 2019; Yang et al., 2018; Shin et al., 2018; Klug et al., 2018; Fernández Menéndez et al., 2018; Camacho-Cardenosa et al., 2018a; Park et al., 2017; Kong et al., 2017; Zhao and Shi, 2016; Nishiwaki et al., 2016; Gutwenger et al., 2015; Gatterer et al., 2015; Morishima et al., 2014; Kong et al., 2014; Li et al., 2014; Wiesner et al., 2010) was performed, 499 men and 480 women, Age: 40.25 ± 15.69, BMI: 30.96 ± 3.65; The seven studies (Han, 2020; Yan, 2020; Mai et al., 2020; Camacho-Cardenosa et al., 2019; De Groote et al., 2018; Camacho-Cardenosa et al., 2018b; Netzer et al., 2008) for which raw data could not be extracted have been analyzed descriptively in Supplementary Table 2), four specified allocation concealment. In terms of blind evaluation, 13 studies were single-blind and six studies were double-blind, and three of them informed the subjects of the entire trial process and risks. Eight of the studies had problems with participants dropping out of the trial during the intervention. The evaluation results are depicted in Figure 2.

### 3.3 Certainty of evidence

The overall certainty of evidence underwent assessment with the application of the GRADE tool, and the findings are presented within Supplementary Table 3. The GRADE method demonstrated that the certainty levels for BM, BFR, TC and FBG were low, while those for BMI, TG, LDL - C, HDL - C, BFI and HOMA - IR were very low.

### 3.4 Meta-analysis

#### 3.4.1 Body mass

**FIGURE 3:**
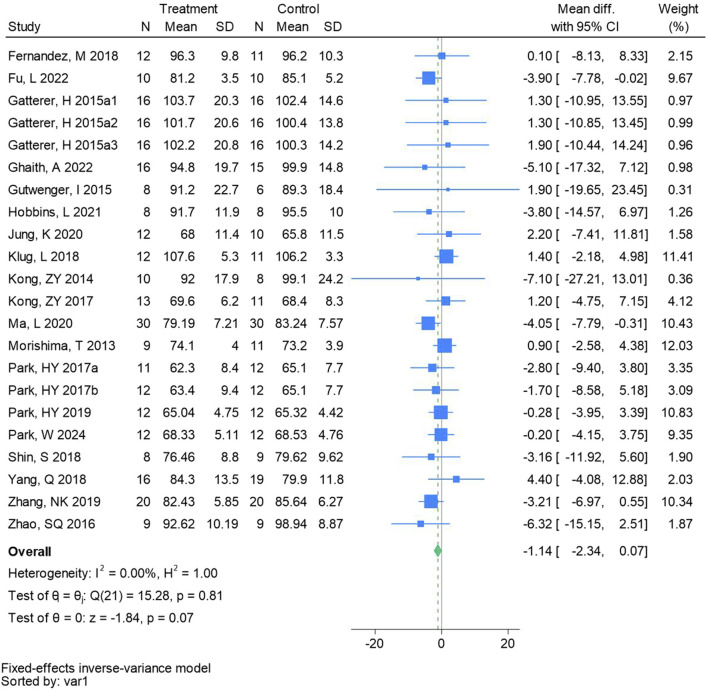
Forest plot of body mass meta-analysis. Gatterer, H 2015a1 indicated that the intervention period was 5 weeks, 2015a2 was 3 months, and 2015a3 was 8 months (Park et al., 2017). indicates training in a hypoxia environment at an altitude of 2,000 m, and 2017b indicates training in a hypoxia environment at an altitude of 3,000 m.

Twenty-two randomized controlled trials were utilized to assess the body mass (See Figure 3 for individual studies), there was no significant difference in the improvement of the body mass between the hypoxic group and the control group and no heterogeneity among the studies (MD -1.14, 95% CI -2.34 to −0.07; *P* = 0.07, *I* ^2^ = 0%).

#### 3.4.2 Body fat ratio

**FIGURE 4:**
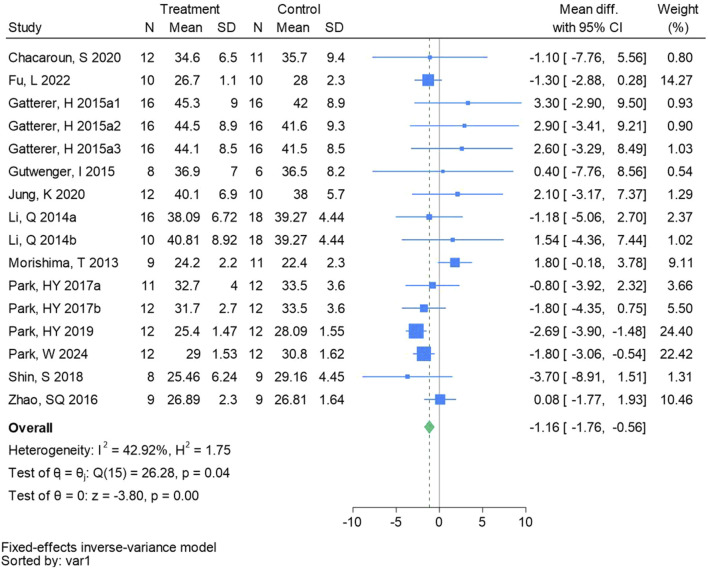
Forest plot of body fat ratio meta-analysis. Gatterer, H 2015a1 indicated that the intervention period was 5 weeks, 2015a2 was 3 months, and 2015a3 was 8 months (Park et al., 2017). indicates training in a hypoxia environment at an altitude of 2,000 m, and 2017b indicates training in a hypoxia environment at an altitude of 3,000 m.

Sixteen randomized controlled trials were included in the analysis of body fat ratio (See Figure 4 for individual studies), there was a significant difference in the improvement of body fat ratio between the hypoxic group and the control group and no heterogeneity among the studies (MD -1.16, 95% CI -1.76 to −0.56; *P* = 0.0001, *I* ^2^ = 42.92%).

#### 3.4.3 Body mass index

**FIGURE 5:**
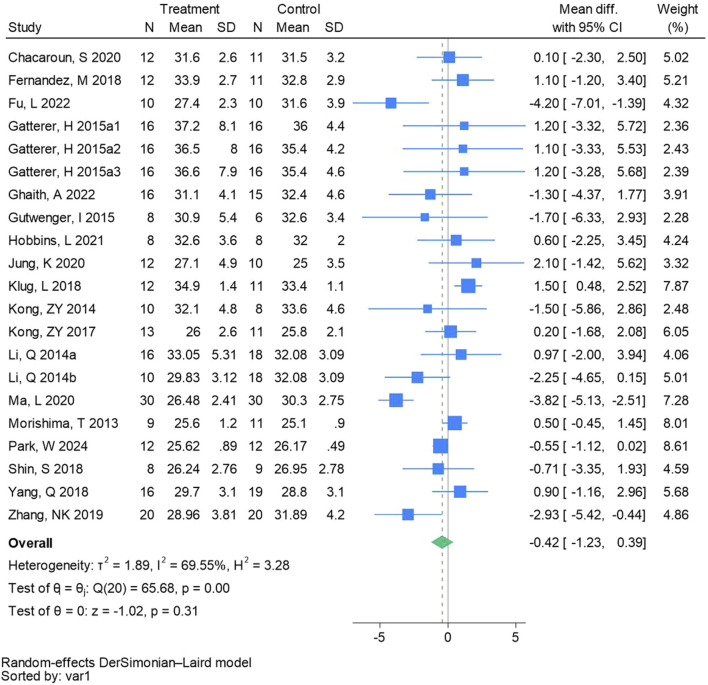
Forest plot of body mass index meta-analysis. Gatterer, H 2015a1 indicated that the intervention period was 5 weeks, 2015a2 was 3 months, and 2015a3 was 8 months (Li et al., 2014). represents the normobaric hypoxia environment, and 2014b represents the hypobaric hypoxia environment.

Twenty-one randomized controlled trials were included in the analysis of BMI (See Figure 5 for individual studies), there was no significant difference in the improvement of BMI between the hypoxic group and the control group and heterogeneity among the studies (MD -0.42, 95% CI -1.23 to 0.39; *P* = 0.31, *I* ^2^ = 69.55%).

#### 3.4.4 Total cholesterol

**FIGURE 6:**
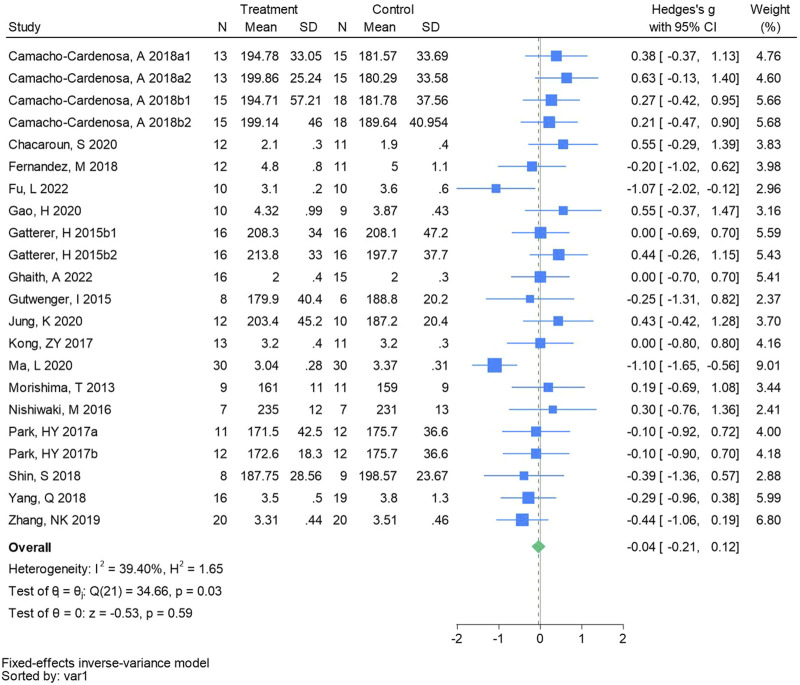
Forest plot of total cholesterol meta-analysis. Camacho-Cardenosa et al., 2018a stands for high-intensity interval training, 2018b stands for high intensity full sprint (1 means that the intervention period is 6 weeks, two means that the intervention period is 12 weeks). Gatterer, H 2015b1 indicated that the intervention period was 3 months, and 2015b2 indicated that the intervention period was 8 months (Park et al., 2017). indicates training in a hypoxia environment at an altitude of 2,000 m, and 2017b indicates training in a hypoxia environment at an altitude of 3,000 m.

Twenty-two randomized controlled trials were utilized to analyze total cholesterol (See Figure 6 for individual studies), there was no significant difference in the improvement of total cholesterol between the hypoxic group and the control group and no heterogeneity among the studies (SMD -0.04, 95% CI -0.21 to 0.12; *P* = 0.59, *I* ^2^ = 39.40%).

#### 3.4.5 Triglycerides

**FIGURE 7:**
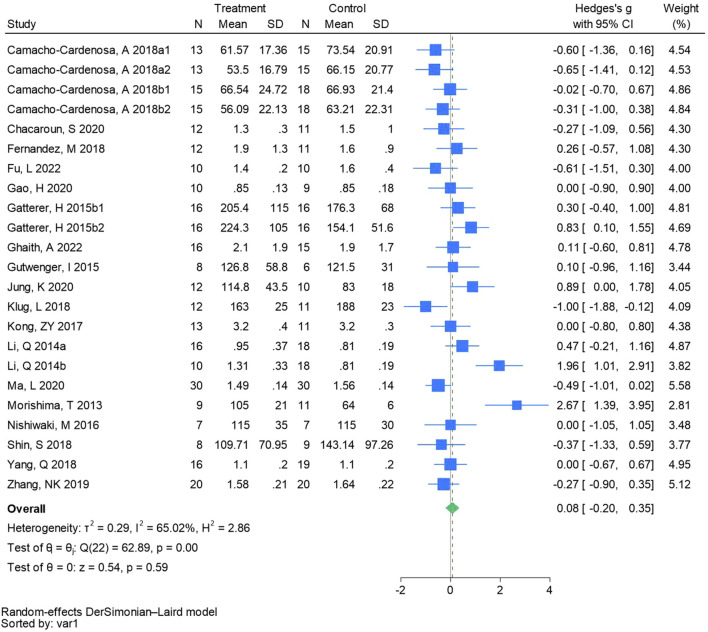
Forest plot of triglycerides meta-analysis. Camacho-Cardenosa et al., 2018a stands for high-intensity interval training, 2018b stands for high intensity full sprint (1 means that the intervention period is 6 weeks, two means that the intervention period is 12 weeks). Gatterer, H 2015b1 indicated that the intervention period was 3 months, and 2015b2 indicated that the intervention period was 8 months (Li et al., 2014). represents the normobaric hypoxia environment, and 2014b represents the hypobaric hypoxia environment.

Twenty-three randomized controlled trials were utilized to analyze triglycerides (See Figure 7 for individual studies), there was no significant difference in the improvement of triglycerides between the hypoxic group and the control group and heterogeneity among the studie (SMD 0.08, 95% CI -0.20 to 0.35; *P* = 0.59, *I* ^2^ = 65.02%).

#### 3.4.6 Low-density lipoprotein cholesterol

**FIGURE 8:**
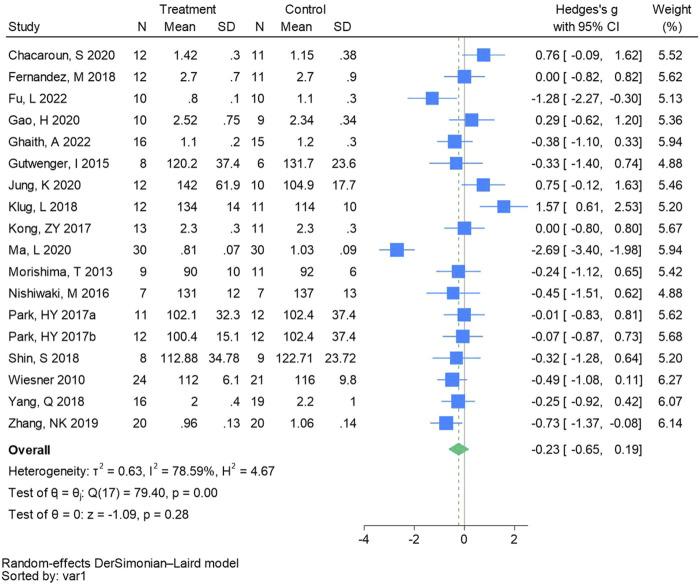
Forest plot of low-density lipoprotein cholesterol meta-analysis (Park et al., 2017). indicates training in a hypoxia environment at an altitude of 2,000 m, and 2017b indicates training in a hypoxia environment at an altitude of 3,000 m.

Eighteen randomized controlled trials were utilized to analyze low-density lipoprotein cholesterol (See Figure 8 for individual studies), there was no significant difference in the improvement of low-density lipoprotein cholesterol between the hypoxic group and the control group and heterogeneity among the studies (SMD -0.23, 95% CI -0.65 to 0.19; *P* = 0.28, *I* ^2^ = 78.59%).

#### 3.4.7 High-density lipoprotein cholesterol

**FIGURE 9:**
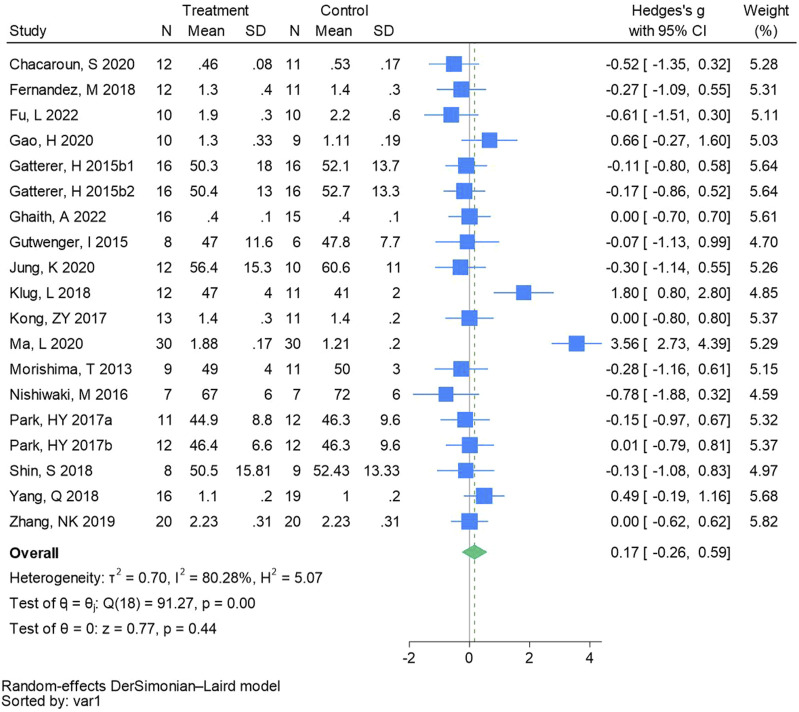
Forest plot of high-density lipoprotein cholesterol meta-analysis. Gatterer, H 2015b1 indicated that the intervention period was 3 months, and 2015b2 indicated that the intervention period was 8 months (Park et al., 2017). indicates training in a hypoxia environment at an altitude of 2,000 m, and 2017b indicates training in a hypoxia environment at an altitude of 3,000 m.

Nineteen randomized controlled trials were employed to analyze high-density lipoprotein cholesterol (See Figure 9 for individual studies), there was no significant difference in the improvement of high-density lipoprotein cholesterol between the hypoxic group and the control group and heterogeneity among the studies (SMD 0.17, 95% CI -0.26 to 0.59; *P* = 0.44, *I* ^2^ = 80.28%).

#### 3.4.8 Fasting blood glucose

**FIGURE 10:**
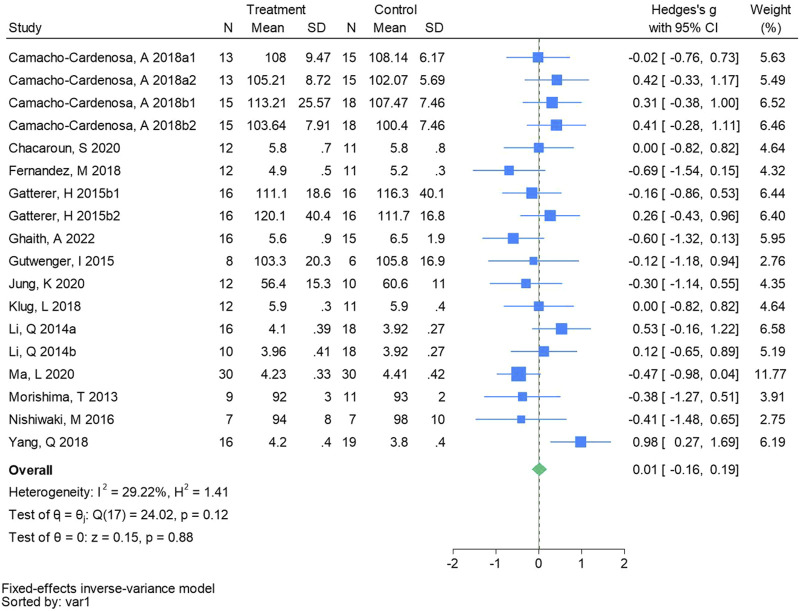
Forest plot of fasting blood glucose meta-analysis. Camacho-Cardenosa et al., 2018a stands for high-intensity interval training, 2018b stands for high intensity full sprint (1 means that the intervention period is 6 weeks, two means that the intervention period is 12 weeks). Gatterer, H 2015b1 indicated that the intervention period was 3 months, and 2015b2 indicated that the intervention period was 8 months (Li et al., 2014). represents the normobaric hypoxia environment, and 2014b represents the hypobaric hypoxia environment.

Eighteen randomized controlled trials were included in the analysis of glucose (See Figure 10 for individual studies), there was no significant difference in the improvement of glucose between the hypoxic group and the control group and no heterogeneity among the studies (SMD 0.01, 95% CI -0.16 to 0.19; *P* = 0.88, *I* ^2^ = 29.22%).

#### 3.4.9 Fasting blood insulin

**FIGURE 11:**
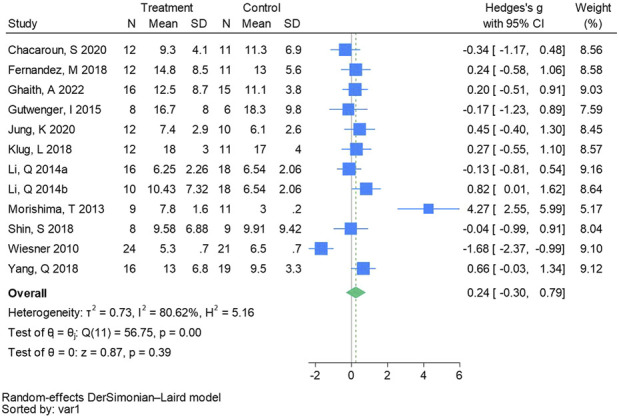
Forest plot of fasting blood insulin meta-analysis (Li et al., 2014). represents the normobaric hypoxia environment, and 2014b represents the hypobaric hypoxia environment.

Twelve randomized controlled trials were included in the analysis of fasting blood insulin (See Figure 11 for individual studies), there was no significant difference in the improvement of fasting blood insulin between the hypoxic group and the control group and heterogeneity among the studies (SMD 0.24, 95% CI -0.30 to 0.79; *P* = 0.39, *I* ^2^ = 80.62%).

#### 3.4.10 Homeostatic assessment of insulin resistance

**FIGURE 12:**
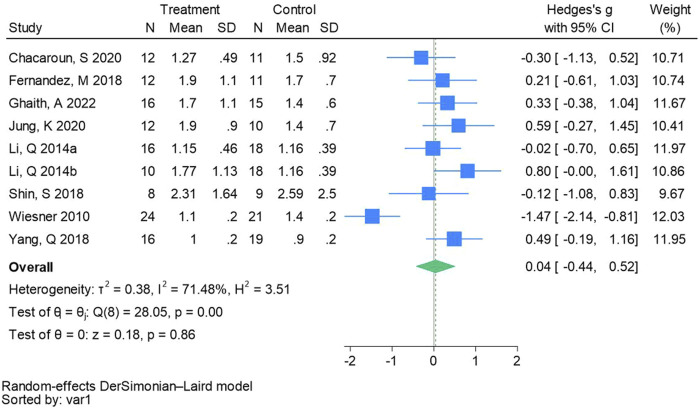
Forest plot of homeostatic assessment of insulin resistance meta-analysis (Li et al., 2014). represents the normobaric hypoxia environment, and 2014b represents the hypobaric hypoxia environment.

Nine randomized controlled trials were included in the analysis of homeostatic assessment of insulin resistance (See Figure 12 for individual studies), there was no significant difference in the improvement of homeostatic assessment of insulin resistance between the hypoxic group and the control group and heterogeneity among the studies (SMD 0.04, 95% CI -0.44 to 0.52; *P* = 0.86, *I* ^2^ = 71.48%).

### 3.5 Sensitivity analysis

Sensitivity analysis of outcome indexes including BMI, TG, LDL - C, HDL - C, FBI, and HOMA - IR was performed. After deleting the literature with high heterogeneity, there were no significant differences in BMI (MD -0.06, 95% CI -0.43 to 0.31; *P* = 0.74, *I* ^2^ = 48%), TG (SMD -0.09, 95% CI -0.25 to 0.08; *P* = 0.30, *I* ^2^ = 30%), LDL - C (SMD -0.20, 95% CI -0.40 to 0.00; *P* = 0.06, *I* ^2^ = 26%), HDL - C (SMD -0.02, 95% CI -0.21 to 0.17; *P* = 0.81, *I* ^2^ = 27%), FBI (SMD 0.22, 95% CI -0.03 to 0.47; *P* = 0.09, *I* ^2^ = 0%) and HOMA - IR (SMD 0.26, 95% CI -0.02 to 0.53; *P* = 0.07, *I* ^2^ = 0%) between the hypoxia group and the normal-oxygen group (Supplementary Table 4).

### 3.6 Subgroup analysis

The effects of hypoxic training on excess body weight or obese people may be influenced by duration, frequency, time, exercise intensity, intervention form, and altitude (Tables 1, 2).

(1) Compared with normoxic training, when the duration of hypoxic training ≤8 weeks, body mass (MD -1.51, 95% CI -2.89 to −0.13; *P* = 0.03, *I* ^2^ = 0%) and LDL - C (SMD -0.25, 95% CI -0.46 to −0.04; *P* = 0.02, *I* ^2^ = 9%) could be effectively improved.
(2) Compared with normoxic training, when the frequency of hypoxic training ≥4 days, body mass (MD -2.70, 95% CI -4.43 to −0.97; *P* = 0.002, *I* ^2^ = 0%), TC (SMD -0.36, 95% CI -0.59 to −0.12; *P* = 0.003, *I* ^2^ = 39%) and LDL - C (SMD -0.28, 95% CI -0.54 to −0.02; *P* = 0.04, *I* ^2^ = 1%) can be effectively improved.
(3) Compared with normoxic training, when the time is 45–60 min of hypoxic training, body mass (MD -1.42, 95% CI -2.70 to −0.15; *P* = 0.03, *I* ^2^ = 0%), TG (SMD -0.27, 95% CI -0.54 to −0.01; *P* = 0.04, *I* ^2^ = 42%) and FBG (SMD -0.34, 95% CI -0.64 to −0.03; *P* = 0.03, *I* ^2^ = 0%) can be effectively improved.
(4) Compared with normoxic training, when the hypoxic training is moderate intensity, body mass (MD -2.29, 95% CI -3.83 to −0.76; *P* = 0.003, *I* ^2^ = 0%), BFR (MD -1.53, 95% CI -2.18 to −0.88; *P* < 0.00001, *I* ^2^ = 31%) and BMI (MD -0.69, 95% CI -1.21 to −0.17; *P* = 0.009, *I* ^2^ = 41%) can be effectively improved.
(5) Compared with normoxic training, when the intervention form of hypoxic training is a combination of aerobic training and resistance training, BFR (MD -2.26, 95% CI -3.14 to −1.39; *P* < 0.00001, *I* ^2^ = 0%) can be effectively improved.
(6) Compared with normoxic training, when the altitude is between 2,001 m and 2,500 m, the body mass (MD -2.09, 95% CI -3.83 to −0.35; *P* = 0.02, *I* ^2^ = 18%), TG (SMD -0.30, 95% CI -0.51 to −0.09; *P* = 0.005, *I* ^2^ = 3%) and LDL - C (SMD -0.42, 95% CI -0.79 to −0.05; *P* = 0.03, *I* ^2^ = 45%) can be effectively improved.

**TABLE 1:** Results of subgroup analysis of duration, frequency and time.

Outcome	Duration (wk)						Frequency (d/wk)						Time (min)					
		n	MD/SMD (95%CI)	*P*	I^2^	*P* (I^2^)		n	MD/SMD (95%CI)	*P*	I^2^	*P* (I^2^)		n	MD/SMD (95%CI)	*P*	I^2^	*P* (I^2^)
BM	≤8	17	−1.51 [-2.89, −0.13]	0.03	0	0.62	<4	10	0.37 [-1.32, 2.06]	0.67	0	0.99	<45	2	−0.01 [-5.36, 5.34]	1	0	0.36
	>8	5	0.07 [-2.41, 2.55]	0.96	0	0.99	≥4	12	−2.70 [-4.43, −0.97]	0.002	0	0.81	45–60	15	−1.42 [-2.70, −0.15]	0.03	0	0.62
													>60	5	2.64 [-2.63, 7.91]	0.33	0	0.99
BFR	≤8	11	−0.36 [-1.20, 0.49]	0.41	9	0.36	<4	9	−0.29 [-2.03, 1.44]	0.74	65	0.003	<45	0				
	>8	5	−1.24 [-2.90, 0.43]	0.14	52	0.08	≥4	7	−0.83 [-1.80, 0.14]	0.09	0	0.86	45–60	10	−1.03 [-2.08, 0.03]	0.06	55	0.02
													>60	6	1.08 [-1.21, 3.37]	0.36	0	0.79
BMI	≤8	17	−0.62 [-1.65, 0.41]	0.24	74	<0.00001	<4	10	0.08 [-0.34, 0.49]	0.72	43	0.07	<45	2	−0.21 [-1.81, 1.40]	0.8	0	0.41
	>8	4	−0.43 [-0.99, 0.13]	0.13	4	0.37	≥4	11	−1.15 [-2.54, 0.24]	0.1	72	0.0001	45–60	11	−0.16 [-1.08, 0.75]	0.72	66	0.0009
													>60	7	0.04 [-1.14, 1.22]	0.95	0	0.45
TC	≤8	17	−0.17 [-0.36, 0.02]	0.08	39	0.05	<4	11	0.26 [0.03, 0.49]	0.03	0	0.91	<45	7	0.25 [-0.04, 0.54]	0.09	0	0.92
	>8	5	0.03 [-0.00, 0.65]	0.05	0	0.79	≥4	11	−0.36 [-0.59, −0.12]	0.003	39	0.09	45–60	10	−0.25 [-0.62, 0.12]	0.18	54	0.02
													>60	5	0.08 [-0.26, 0.43]	0.64	0	0.46
TG	≤8	16	−0.17 [-0.36, 0.02]	0.08	0	0.56	<4	11	−0.09 [-0.42, 0.25]	0.61	53	0.02	<45	7	−0.22 [-0.50, 0.07]	0.14	0	0.7
	>8	5	0.20 [-0.38, 0.78]	0.51	67	0.02	≥4	10	−0.10 [-0.34, 0.13]	0.4	0	0.58	45–60	8	−0.27 [-0.54, −0.01]	0.04	42	0.1
													>60	6	0.31 [0.00, 0.62]	0.05	0	0.61
LDL - C	≤8	15	−0.25 [-0.46, −0.04]	0.02	9	0.35	<4	6	−0.02 [-0.49, 0.44]	0.93	51	0.07	<45	3	−0.26 [-0.74, 0.21]	0.28	0	0.73
	>8	1	0.75 [-0.12, 1.63]				≥4	10	−0.28 [-0.54, −0.02]	0.04	1	0.43	45–60	10	−0.17 [-0.53, 0.19]	0.35	51	0.03
													>60	3	−0.11 [-0.59, 0.37]	0.64	0	0.58
HDL - C	≤8	15	0.02 [-0.20, 0.23]	0.88	38	0.07	<4	8	−0.00 [-0.43, 0.42]	0.99	54	0.03	<45	3	−0.15 [-0.62, 0.33]	0.55	0	0.46
	>8	3	−0.18 [-0.60, 0.24]	0.41	0	0.94	≥4	10	−0.01 [-0.27, 0.24]	0.91	0	0.52	45–60	10	−0.08 [-0.34, 0.18]	0.54	46	0.05
													>60	5	0.14 [-0.20, 0.49]	0.42	0	0.45
FBG	≤8	13	−0.04 [-0.25, 0.17]	0.69	40	0.07	<4	11	0.02 [-0.21, 0.25]	0.86	0	0.64	<45	6	0.07 [-0.24, 0.38]	0.67	21	0.27
	>8	5	0.15 [-0.18, 0.47]	0.37	0	0.56	≥4	7	0.01 [-0.46, 0.49]	0.96	63	0.01	45–60	6	−0.34 [-0.64, −0.03]	0.03	0	0.81
													>60	6	0.31 [0.01, 0.62]	0.04	23	0.26
BFI	≤8	9	0.20 [-0.07, 0.46]	0.14	0	0.5	<4	5	0.12 [-0.25, 0.49]	0.52	0	0.72	<45	1	0.20 [-0.51, 0.91]			
	>8	1	0.45 [-0.40, 1.30]				≥4	5	0.31 [-0.04, 0.66]	0.08	20	0.29	45–60	4	0.15 [-0.26, 0.57]	0.48	0	0.57
													>60	5	0.28 [-0.08, 0.63]	0.13	26	0.25
HOMA - IR	≤8	7	0.22 [-0.07, 0.51]	0.14	0	0.51	<4	3	0.21 [-0.25, 0.66]	0.37	15	0.31	<45	1	0.33 [-0.38, 1.04]			
	>8	1	0.59 [-0.27, 1.45]				≥4	5	0.29 [-0.06, 0.63]	0.1	0	0.48	45–60	3	0.15 [-0.33, 0.63]	0.54	9	0.33
													>60	4	0.30 [-0.08, 0.68]	0.12	13	0.33

Note: BM, body mass; BFR, body fat ratio; BMI, body mass index; TC, total cholesterol; TG, triglyceride; LDL - C, low density lipoprotein cholesterol; HDL - C, high density lipoprotein cholesterol; FNG, fasting blood-glucose; FBI, fasting blood insulin; HOMA - IR, homeostatic assessment of insulin resistance.

**TABLE 2:** Results of subgroup analysis of exercise intensity, intervention form and altitude.

Outcome	Exercise intensity						Intervention form						Altitude (m)					
		n	MD/SMD (95%CI)	*P*	I^2^	*P* (I^2^)		n	MD/SMD (95%CI)	*P*	I^2^	*P* (I^2^)		n	MD/SMD (95%CI)	*P*	I^2^	*P* (I^2^)
BW	LI	8	0.89 [-1.22, 3.01]	0.41	0	0.93	AE	17	−1.43 [-2.83, −0.02]	0.05	0	0.65	1,500–2000	2	−2.40 [-8.71, 3.92]	0.46	0	0.68
	MI	12	−2.29 [-3.83, −0.76]	0.003	0	0.86	HE	2	−0.01 [-5.36, 5.34]	1	0	0.36	2001–2,500	7	−2.09 [-3.83, −0.35]	0.02	18	0.29
	HI	2	−0.01 [-5.36, 5.34]	1	0	0.36	CE	3	−0.36 [-3.03, 2.30]	0.79	0	0.80	2,501–3,000	8	0.03 [-1.81, 1.88]	0.97	0	0.81
													>3,000	5	−1.05 [-6.36, 4.27]	0.7	0	0.88
BFR	LI	6	0.85 [-0.66, 2.36]	0.27	3	0.4	AE	14	−0.19 [-1.00, 0.63]	0.66	5	0.4	1,500–2000	2	−0.65 [-3.56, 2.27]	0.66	0	0.79
	MI	10	−1.53 [-2.18, −0.88]	<0.00001	31	0.16	HE	0					2001–2,500	4	−1.30 [-2.67, 0.07]	0.06	0	0.63
	HI	0					CE	2	−2.26 [-3.14, −1.39]	<0.00001	0	0.32	2,501–3,000	5	−0.96 [-2,51, 0.59]	0.22	77	0.002
													>3,000	5	2.05 [-0.63, 4.47]	0.13	0	0.89
BMI	LI	10	0.60 [-0.10, 1.30]	0.09	20	0.26	AE	16	0.36 [-0.15, 0.88]	0.17	50	0.01	1,500–2000	1	−1.70 [-6.33, 2.93]			
	MI	8	−0.69 [-1.21, −0.17]	0.009	41	0.1	HE	2	−0.21 [-1.81, 1.40]	0.8	0	0.41	2001–2,500	9	−1.38 [-3.14, 0.37]	0.12	85	<0.00001
	HI	2	−0.21 [-1.81, 1.40]	0.8	0	0.41	CE	2	−0.57 [-1.14, 0.00]	0.05	0	0.67	2,501–3,000	5	−0.11 [-0.58, 0.35]	0.64	46	0.12
													>3,000	6	0.23 [-1.11, 1.58]	0.74	0	0.91
TC	LI	8	0.02 [-0.29, 0.33]	0.92	0	0.7	AE	16	−0.17 [-0.37, 0.03]	0.09	45	0.03	1,500–2000	3	−0.03 [-0.58, 0.52]	0.91	0	0.75
	MI	8	−0.24 [-0.68, 0.20]	0.29	66	0.005	HE	6	0.25 [-0.05, 0.64]	0.10	0	0.86	2001–2,500	10	−0.10 [-0.51, 0.31]	0.62	67	0.001
	HI	6	0.25 [-0.05, 0.64]	0.10	0	0.86	CE	0					2,501–3,000	5	−0.03 [-0.39, 0.32]	0.85	0	0.71
													>3,000	4	0.22 [-0.14, 0.59]	0.23	0	0.63
TG	LI	9	0.07 [-0.21, 0.35]	0.61	30	0.18	AE	15	−0.02 [-0.22, 0.17]	0.81	41	0.05	1,500–2000	2	0.05 [-0.70, 0.79]	0.9	0	0.89
	MI	6	−0.09 [-0.52, 0.34]	0.69	56	0.04	HE	6	−0.23 [-0.53, 0.07]	0.13	0	0.60	2001–2,500	12	−0.30 [-0.51, −0.09]	0.005	3	0.41
	HI	6	−0.23 [-0.53, 0.07]	0.13	0	0.60	CE	0					2,501–3,000	3	0.30 [-0.15, 0.75]	0.19	19	0.29
													>3,000	4	0.27 [-0.10, 0.64]	0.15	27	0.25
LDL - C	LI	8	−0.05 [-0.36, 0.26]	0.74	0	0.61	AE	14	−0.19 [-0.41, 0.02]	0.08	34	0.10	1,500–2000	3	−0.22 [-0.77, 0.34]	0.45	0	0.79
	MI	6	−0.30 [-0.80, 0.19]	0.23	61	0.03	HE	2	−0.22 [-0.75, 0.32]	0.43	0	0.48	2001–2,500	5	−0.42 [-0.79, −0.05]	0.03	45	0.12
	HI	2	−0.22 [-0.75, 0.32]	0.43	0	0.48	CE	0					2,501–3,000	6	−0.13 [-0.43, 0.18]	0.41	11	0.35
													>3,000	2	0.09 [-0.46, 0.63]	0.76	76	0.04
HDL - C	LI	9	0.13 [-0.33, 0.60]	0.58	58	0.02	AE	16	−0.03 [-0.23, 0.18]	0.80	36	0.08	1,500–2000	3	−0.29 [-0.85, 0.27]	0.31	0	0.59
	MI	7	−0.18 [-0.46, 0.10]	0.21	0	0.91	HE	2	0.00 [-0.53, 0.53]	1.00	0	1	2001–2,500	6	0.26 [-0.35, 0.86]	0.40	67	0.01
	HI	2	0.00 [-0.53, 0.53]	1.00	0	1	CE	0					2,501–3,000	5	−0.01 [-0.37, 0.35]	0.95	0	0.51
													>3,000	4	−0.17 [-0.54, 0.19]	0.35	0	0.82
FBG	LI	9	0.07 [-0.21, 0.35]	0.62	42	0.09	AE	13	−0.03 [-0.24, 0.18]	0.79	33	0.12	1,500–2000	2	−0.27 [-1.02, 0.48]	0.48	0	0.70
	MI	4	−0.17 [-0.49, 0.16]	0.31	0	0.4	HE	5	0.11 [-0.21, 0.43]	0.50	27	0.24	2001–2,500	8	0.10 [-0.14, 0.35]	0.40	15	0.31
	HI	5	0.11 [-0.21, 0.43]	0.50	27	0.24	CE	0					2,501–3,000	4	−0.07 [-0.86, 0.71]	0.86	73	0.01
													>3,000	4	−0.02 [-0.42, 0.37]	0.90	0	0.72
BFI	LI	8	0.29 [0.01, 0.58]	0.05	0	0.59	AE	9	0.22 [-0.05, 0.49]	0.11	0	0.47	1,500–2000	1	−0.17 [-1.23, 0.89]			
	MI	1	−0.34 [-1.17, 0.48]				HE	1	0.20 [-0.51, 0.91]				2001–2,500	4	0.21 [-0.19. 0.61]	0.30	13	0.33
	HI	1	0.20 [-0.51, 0.91]				CE	0					2,501–3,000	3	0.48 [0.03, 0.92]	0.04	0	0.74
													>3,000	2	−0.03 [-0.57, 0.51]	0.91	0	0.33
HOMA - IR	LI	6	0.33 [0.01, 0.65]	0.04	0	0.56	AE	7	0.24 [-0.05, 0.54]	0.11	0	0.44	1,500–2000	0				
	MI	1	−0.30 [-1.13, 0.52]				HE	1	0.33 [-0.38, 1.04]				2001–2,500	3	0.22 [-0.24, 0.67]	0.35	34	0.22
	HI	1	0.33 [-0.38, 1.04]				CE	0					2,501–3,000	3	0.43 [-0.01, 0.88]	0.06	0	0.8
													>3,000	2	0.06 [-0.48, 0.60]	0.83	23	0.25

Note: LI, low intensity; MI, medium intensity; HI, high intensity; AE, aerobic exercise; HE, high intensity exercise; CE, combination exercise (aerobic and resistance).

### 3.7 Publication bias analysis

**FIGURE 13:**
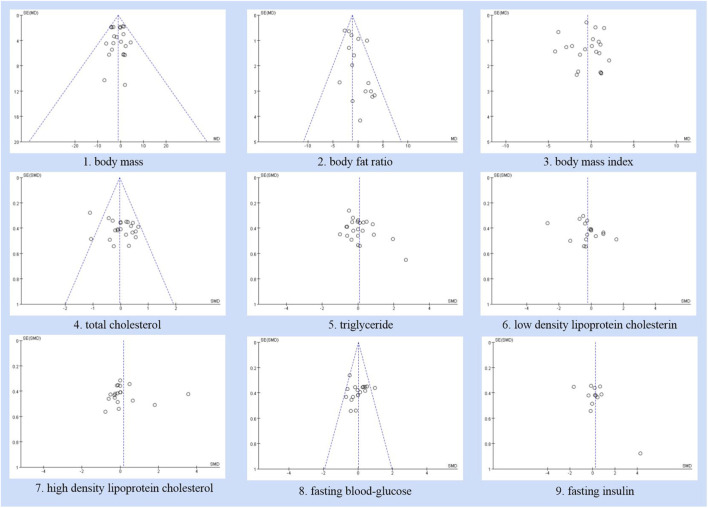
Publication Bias Analysis result.

The funnel plot of body weight results showed reasonable symmetry, while the funnel plot analysis of other outcome measures showed slight asymmetry (Figure 13). In the meta-analysis, funnel plot analysis is not recommended for assessing publication bias when the included literature has fewer than 10 outcome indicators (Chang et al., 2015). Egger’s regression test for HOMA - IR did not reach statistical significance (*P* = 0.04727).

## 4 Discussion

This study aims to assess the impact of hypoxic training on enhancing body composition and glucose and lipid metabolism in excess body weight and with obese people via meta-analysis, aiming to ascertain its efficacy compared to normoxic training. Explore the best hypoxic training program for excess body weight or obese people. The main finding of this meta-analysis was that hypoxic training significantly reduced body fat ratio in excess body weight or living with obese people, but there was no significant difference in glycolipid metabolism. Subgroup analysis showed that training in the hypoxic environment at an altitude of 2001–2500 m could effectively decrease body mass, TG, and LDL - C. The effective program to reduce body mass is to carry out moderate intensity training (MD -2.29, 95% CI -3.83 to −0.76; *P* = 0.003, *I* ^2^ = 0%) of 45–60 min (MD -1.42, 95% CI -2.70 to −0.15; *P* = 0.03, *I* ^2^ = 0%) for ≤8 weeks (MD -1.51, 95% CI -2.89 to −0.13; *P* = 0.03, *I* ^2^ = 0%), ≥4 times a week (MD -2.70, 95% CI -4.43 to −0.97; *P* = 0.002, *I* ^2^ = 0%).

Previous studies have not found that hypoxic training can significantly improve body composition, which may be affected by different training types and populations (Chen et al., 2022; Ramos-Campo et al., 2019; Guo et al., 2023). The results of this meta-analysis revealed a significant superiority of the hypoxia group over the normal-oxygen group in both body fat ratios (MD is −1.16, 95% CI -1.76 to −0.56, *P* = 0.00). It is suggested that hypoxia training can significantly reduce the body fat ratio of excess body weight and obese people, which is more significant in the combination of aerobic and resistance training intervention (MD is −2.26, 95% CI -3.14 to −1.39, *P* = 0.00). A recent meta - analysis has revealed that the integration of aerobic and resistance training leads to significant beneficial changes in multiple cardiometabolic parameters and mental health - associated markers among excess body weight or obese patients (Al-Mhanna et al., 2024). Furthermore, resistance training, when employed as an independent exercise intervention, confers numerous cardiometabolic advantages in the management and treatment of type 2 diabetes mellitus (T2DM) patients who are excess body weight or obese (Al-Mhanna et al., 2025). Despite the weak evidence for hypoxic training on body mass (MD is −1.14, 95% CI -2.34 to 0.07, *P* = 0.07) and BMI (MD is −0.42, 95% CI -1.23 to 0.39, *P* = 0.31) outcomes shown in our meta-analysis. A body of research indicates the efficacy of hypoxia training in improving the body composition of excess body weight and living with obese individuals (Tee et al., 2023; Kong et al., 2014). The combined effect of hypoxia and exercise triggers a stress response in the body, accelerating fat decomposition (Workman and Basset, 2012). Exposure to hypoxia enhanced the ability to transport oxygen to muscle and the improvement of fat oxidation (Park et al., 2016; Chen et al., 2013). Additionally, a hypoxia environment boosts metabolic rate, increases leptin and insulin-like growth factor levels, and stimulates the hematopoietic system, ultimately reducing body fat content (Yingzhong et al., 2006). Subgroup analysis showed that moderate-intensity aerobic exercise (≤8 weeks, ≥4 times/week, 45–60 min) at altitude 2001–2500 m in a hypoxia environment had the greatest improvement in body weight. Hypoxia can inhibit Ghrelin concentration (Bailey et al., 2015), and exercise in a hypoxia environment may lead to a substantial decrease in energy intake. The decrease in energy intake caused by low oxygen exposure and the increase in energy expenditure caused by an increase in basal metabolic rate may be the main causes of weight loss (Millet et al., 2016). The study found that exercise intensity is a crucial factor influencing fatty acids. When engaging in sustained exercise at 25% of the maximum intensity, 90% of the energy is sourced from fatty acid oxidation, 10% from liver glycogen, and the fatty acids primarily originate from the hydrolysis of triglycerides in adipose tissue (Romijn et al., 1993). Therefore, low or moderate exercise intensity is more conducive to fat mobilization. Vascular endothelial growth factor (VEGF) expression, skeletal muscle capillary density increase (Vogt et al., 2001), and myoglobin content significantly increased after hypoxia training, which is conducive to the transfer of more oxygen and fatty acids into skeletal muscle mitochondria to participate in oxidation and energy supply (Zoll et al., 2006), this may be a beneficial mechanism for weight loss. Therefore, for obese people who want to achieve a better weight loss effect in the short term, it is recommended to carry out moderate-intensity aerobic training (≥4 times a week, 45–60 min) in a hypoxia environment at an altitude of 2001–2,500 m, which can effectively reduce weight.

The results of this meta-analysis showed that there were no significant differences in TC (Hedges’s g is −0.04, 95% CI -0.21 to 0.12, *P* = 0.59), TG (Hedges’s g is 0.08, 95% CI -0.20 to 0.35, *P* = 0.59), LDL - C (Hedges’s g is −0.23, 95% CI -0.65 to 0.19, *P* = 0.28), and HDL - C (Hedges’s g is 0.17, 95% CI -0.26 to 0.59, *P* = 0.44) levels between the hypoxic group and the normal oxygen group. This is consistent with the research results of previous scholars (Chen et al., 2022; Guo et al., 2023; Ramos-Campo et al., 2019). Although there is no significant difference, there are many studies showing that hypoxic training is significantly better than normal oxygen training, and these studies are conducted in a hypoxic environment at an altitude of 2,500 m, five times a week, each time 60 min of aerobic exercise. Engaging in regular physical activity not only proves highly effective in boosting HDL - C levels but also brings about a significant reduction in TG, TC, and LDL - C levels, thus contributing comprehensively to better lipid metabolism and overall cardiovascular health (Badri Al-mhanna et al., 2024b). There is ongoing debate regarding the efficacy of hypoxic training in enhancing lipid metabolism among excess body weight and living with obese people. Research has demonstrated that a 4-week regimen of hypoxia training substantially enhances levels of TC, TG, LDL - C, and HDL - C in living with obese rats (Yan, 2020). Regular endurance exercise can induce favorable alterations in blood lipids and lipoproteins, particularly in hypoxia environments. This could be attributed to the combined effects of hypoxia and exercise, leading to an elevation in serum leptin levels, enhanced adipose tissue catabolism (Huan et al., 2008), improved insulin sensitivity, and reduced fat synthesis, ultimately contributing to the enhancement of blood lipid profiles (Wiesner et al., 2010). Elevated LDL - C levels and reduced HDL - C levels also elevate the risk of atherosclerosis (Marso et al., 2008). Subgroup analysis showed that exercise training for ≤8 weeks and ≥4 times per week at an altitude of 2,001–2,005 m was effective in improving LDL - C. Some suggest that the duration of training may impact the improvement of blood lipid levels (Chen et al., 2022). However, following 8 weeks of hypoxia training, there was a greater improvement in lipid metabolism levels among living with obese individuals (Netzer et al., 2017). In excess body weight and obese individuals, regular exercise is beneficial for enhancing HDL - C levels, yet the most effective strategy is a combination of moderate - to - high - intensity, long - term exercise performed at the aerobic threshold, accompanied by dietary changes (Badri Al-mhanna et al., 2024a). Furthermore, numerous studies suggest that exercise has a minimal impact on TG levels in living with obese individuals (Wiesner et al., 2010; Chacaroun et al., 2020; Wang et al., 2013), potentially attributable to TG primarily deriving from fat decomposition in food. A subgroup analysis then showed that 45–60 min of training in a hypoxia environment at an altitude of 2001–2,500 m had a significant effect on TG. Research has demonstrated that fat intake can be diminished by adhering to a low-fat diet (Chiu et al., 2015), eliminating lipid deposition in blood vessels, augmenting vascular formation (Tan et al., 2016), and mitigating the risk of atherosclerosis. However, the present study lacks rigorous dietary control for living with obese individuals, necessitating further investigation to ascertain whether strict dietary regulation could notably improve lipid metabolism. Therefore, we recommend that in a hypoxia environment at an altitude of 2001–2,500 m, develop good exercise habits (≥4 times a week, 45–60 min aerobic exercise) and strictly control diet, which is a better program to control lipid metabolism.

The results of this meta-analysis revealed no significant differences in FBG (Hedges’s g is 0.01, 95% CI -0.16 to 0.19, *P* = 0.88), FBI (Hedges’s g is 0.24, 95% CI -0.30 to 0.79, *P* = 0.39), and HOMA - IR (Hedges’s g is 0.04, 95% CI -0.44 to 0.52, *P* = 0.86) levels within the hypoxic group. This is consistent with the research results of previous scholars (Guo et al., 2023; Chen et al., 2022). Although there were no significant differences, two or three studies included in the analysis showed that both hypoxia and normoxia training boosted glucose metabolism levels. The exercise intervention in one study was 120 min of low-intensity exercise six times a week, and the exercise intervention in the other two studies was 60 min of moderate-intensity exercise 5/6 times a week. The majority of studies indicate that both hypoxia and normal oxygen training can enhance glucose metabolism in living with obese individuals (De Groote et al., 2018; Wiesner et al., 2010). Ounis et al. (2008) reported significant reductions in HOMA - IR among excess body weight and living with obese adolescents following 8 weeks of aerobic training. Excess adiposity severity correlates positively with diabetes incidence attributable to insulin resistance. Adequate physical activity enhances insulin sensitivity and promotes glucose utilization, thereby reducing blood sugar levels, improving glycemic control, and effectively preventing and managing diabetes. Presently, there is a paucity of randomized controlled trials (RCTs) investigating the combined effects of hypoxia and exercise intervention on glucose metabolism among excess body weight and living obese people. These trials are influenced by factors such as hypoxia mode, oxygen concentration, gender, load intensity, sample size, and diet. Research indicates that female youth in the hypoxic group exhibit greater reductions in HOMA - IR (Wang et al., 2013), possibly attributable to accelerated visceral fat decomposition in hypoxic environments, thereby ameliorating metabolic disorders and enhancing insulin sensitivity. Therefore, the effects of hypoxic training on glucose metabolism still need more high-quality literature to explore.

The limitations of this paper include the absence of allocation hiding and blind methods in some of the included literature, along with instances of subject loss, posing a significant risk of bias. Variability exists in the training protocols across the literature, including differences in hypoxia mode, oxygen concentration, and load intensity. In this paper, all age groups were included in the analysis, and the age span of subjects in some studies was too large to conduct a more in-depth analysis of age.

## 5 Conclusion

Hypoxic training is essential for reducing body fat ratio in excess body weight or obese people. It is recommended to carry out 45–60 min of moderate-intensity aerobic exercise for ≤8 weeks, ≥4 times a week, in a hypoxia environment of 2001–2,500 m to lose body mass. The effects of hypoxia training and normoxia training on lipid and glucose metabolism in excess body weight or obese people are the same.

## Data Availability

The original contributions presented in the study are included in the article/Supplementary Material, further inquiries can be directed to the corresponding author.
