# Supplementary material for: Impact of exercise training in a hypobaric/normobaric hypoxic environment on body composition and glycolipid metabolism in individuals with overweight or obesity: a systematic review and meta-analysis
Source: Front Physiol. 2025 Mar 10;16:1571730. doi: 10.3389/fphys.2025.1571730 (PMC11931047; doi:10.3389/fphys.2025.1571730)
Supplement: Supplementary file 1 [file Table1.docx]

Supplementary Material

Supplementary Table 1. Search strategy

| Databases | Search strategy | Results |
| --- | --- | --- |
| Pub Med | (("Intermittent hypoxia" OR "hypoxia exercise" OR "hypoxic training" OR "altitude training" OR "normobaric hypoxia training") AND ("overweight" OR "obese" OR "obesity")) | 394 |
| Web of science | (TS=("Intermittent hypoxia" OR "hypoxia exercise" OR "hypoxic training" OR "altitude training" OR "normobaric hypoxia training")) AND TS=("overweight" OR "obese" OR "obesity") | 991 |
| China National Knowledge Infrastructure | ("Intermittent hypoxia" + "hypoxia exercise" + "hypoxic training" + "altitude training" + "normobaric hypoxia training" + "hypoxia") AND ("overweight" + "obese" + "obesity") | 289 |
