# Supplementary figures and images for: Impact of exercise training in a hypobaric/normobaric hypoxic environment on body composition and glycolipid metabolism in individuals with overweight or obesity: a systematic review and meta-analysis
Source: Front Physiol. 2025 Mar 10;16:1571730. doi: 10.3389/fphys.2025.1571730 (PMC11931047; doi:10.3389/fphys.2025.1571730)

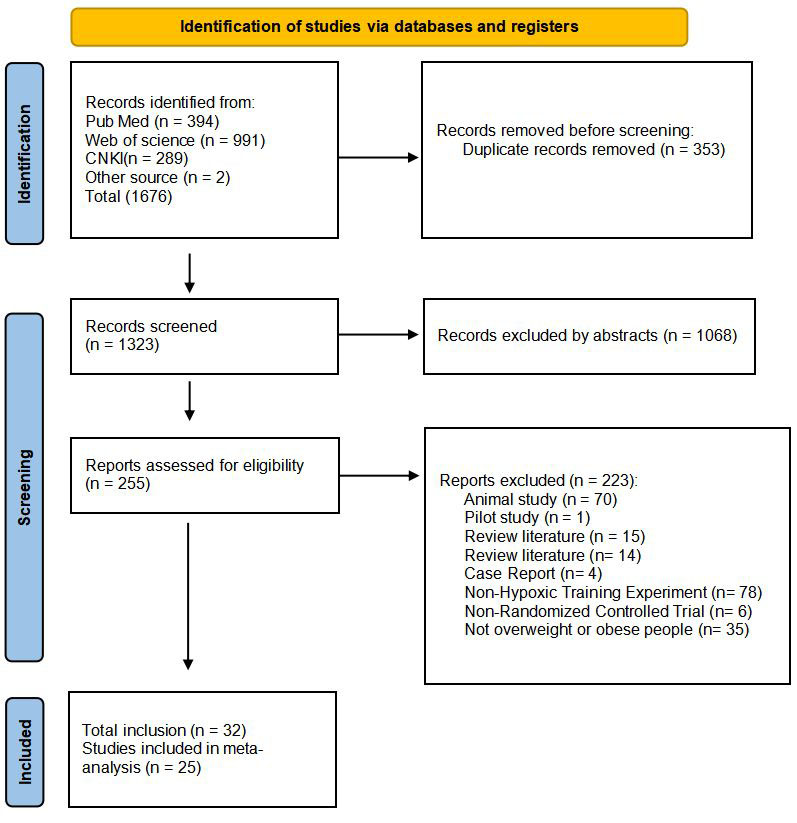

Supplement: Supplementary file 2 [file DataSheet1.zip › Supplementary Material Presentation/Figure 1.jpeg]

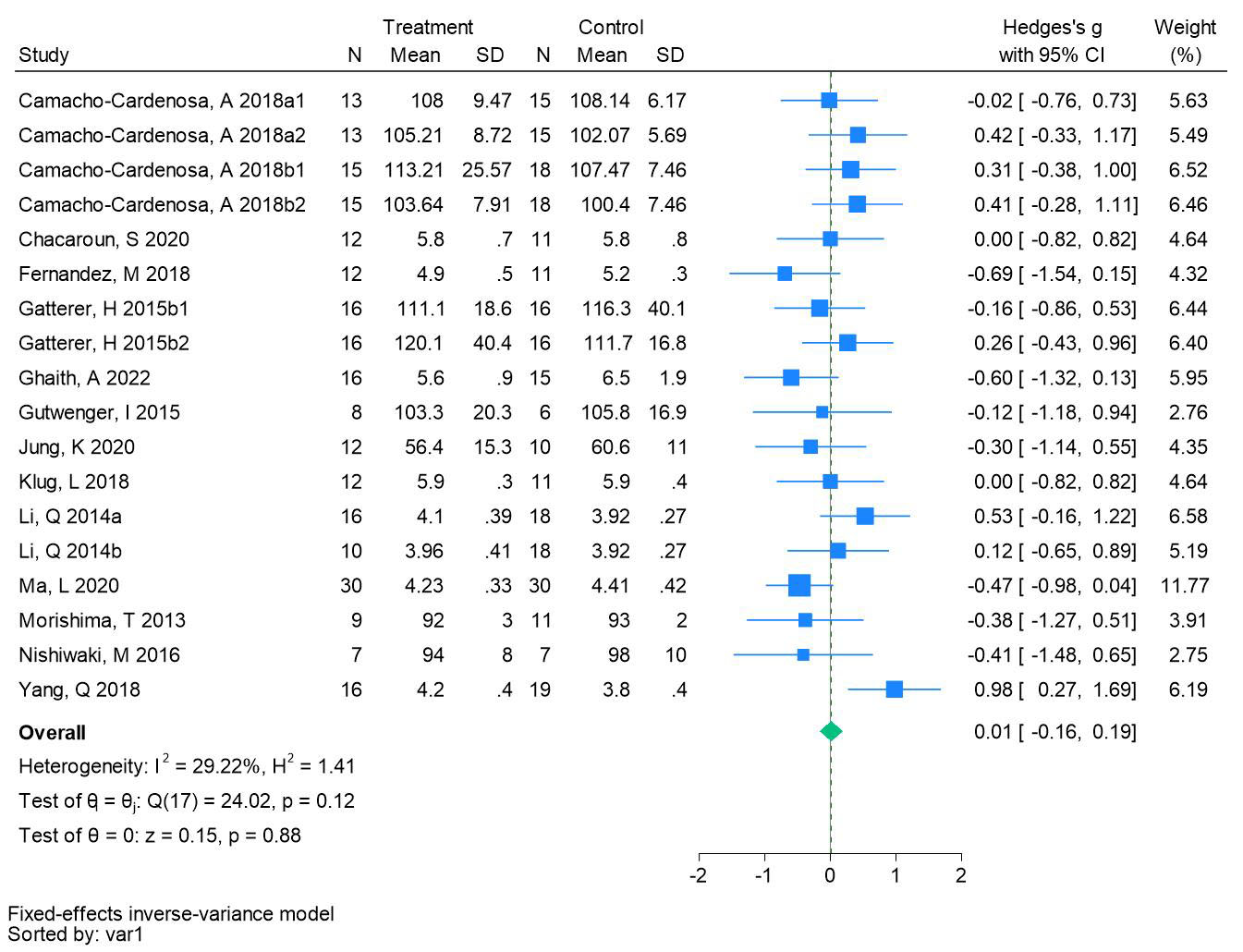

Supplement: Supplementary file 2 [file DataSheet1.zip › Supplementary Material Presentation/Figure 10.jpeg]

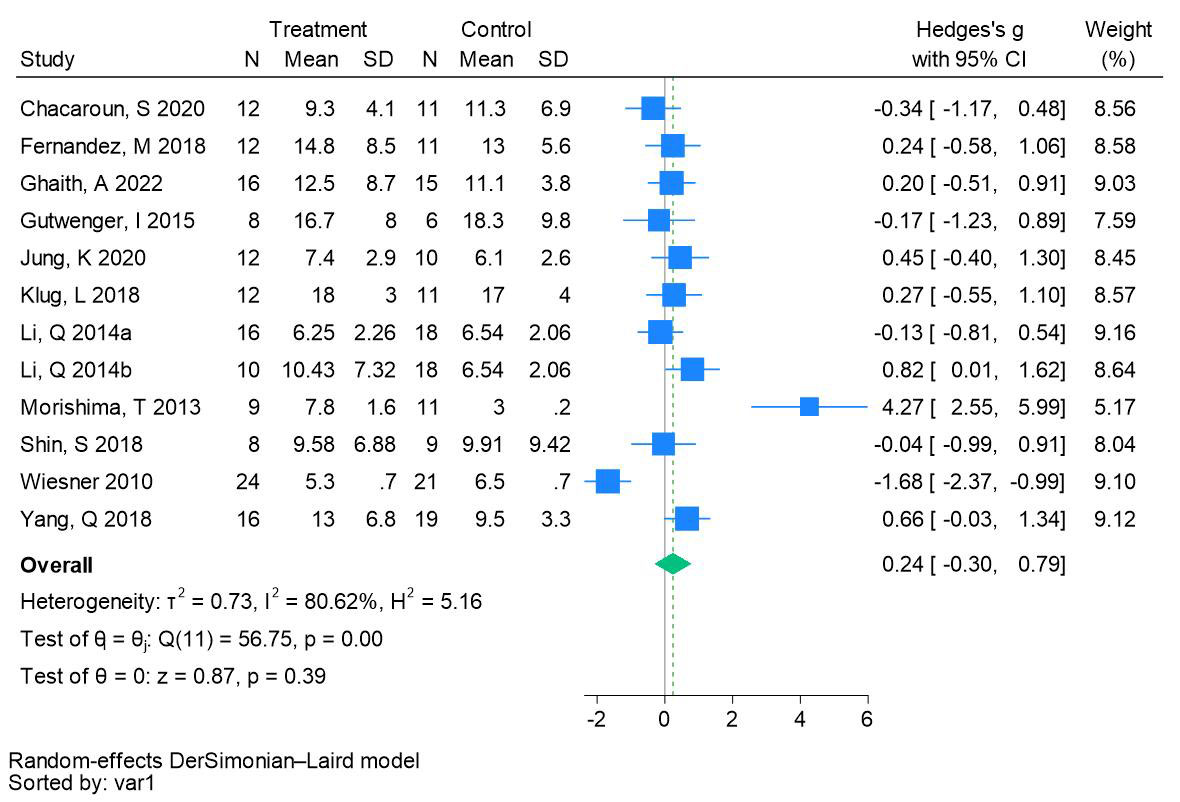

Supplement: Supplementary file 2 [file DataSheet1.zip › Supplementary Material Presentation/Figure 11.jpeg]

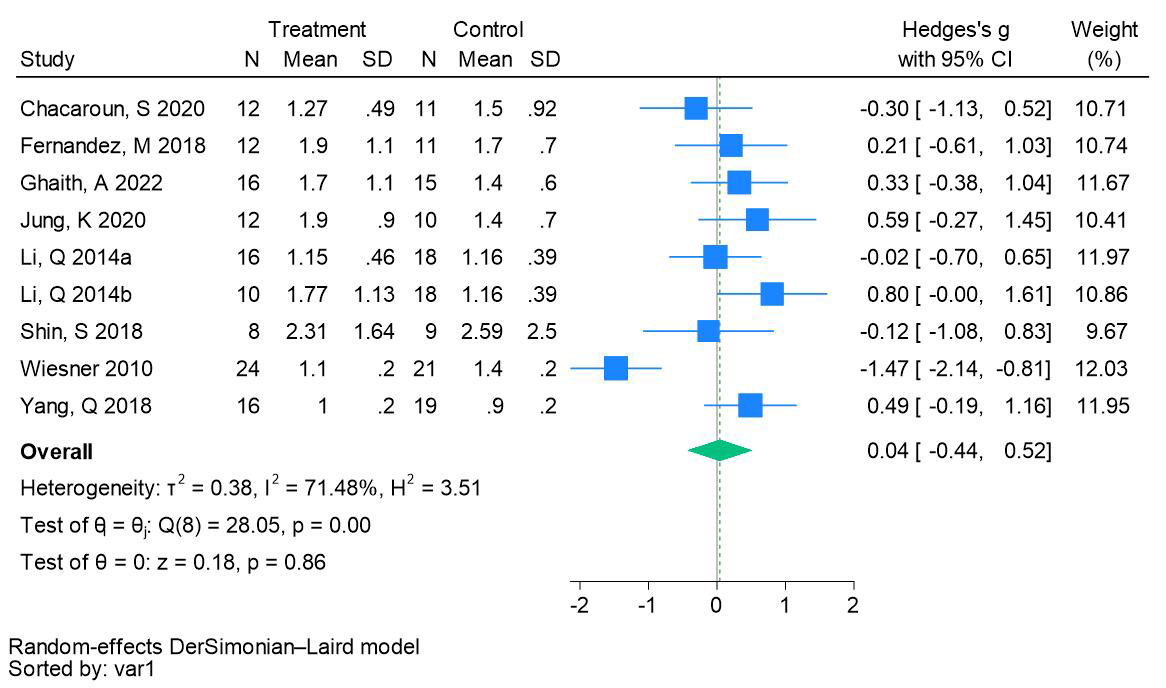

Supplement: Supplementary file 2 [file DataSheet1.zip › Supplementary Material Presentation/Figure 12.jpeg]

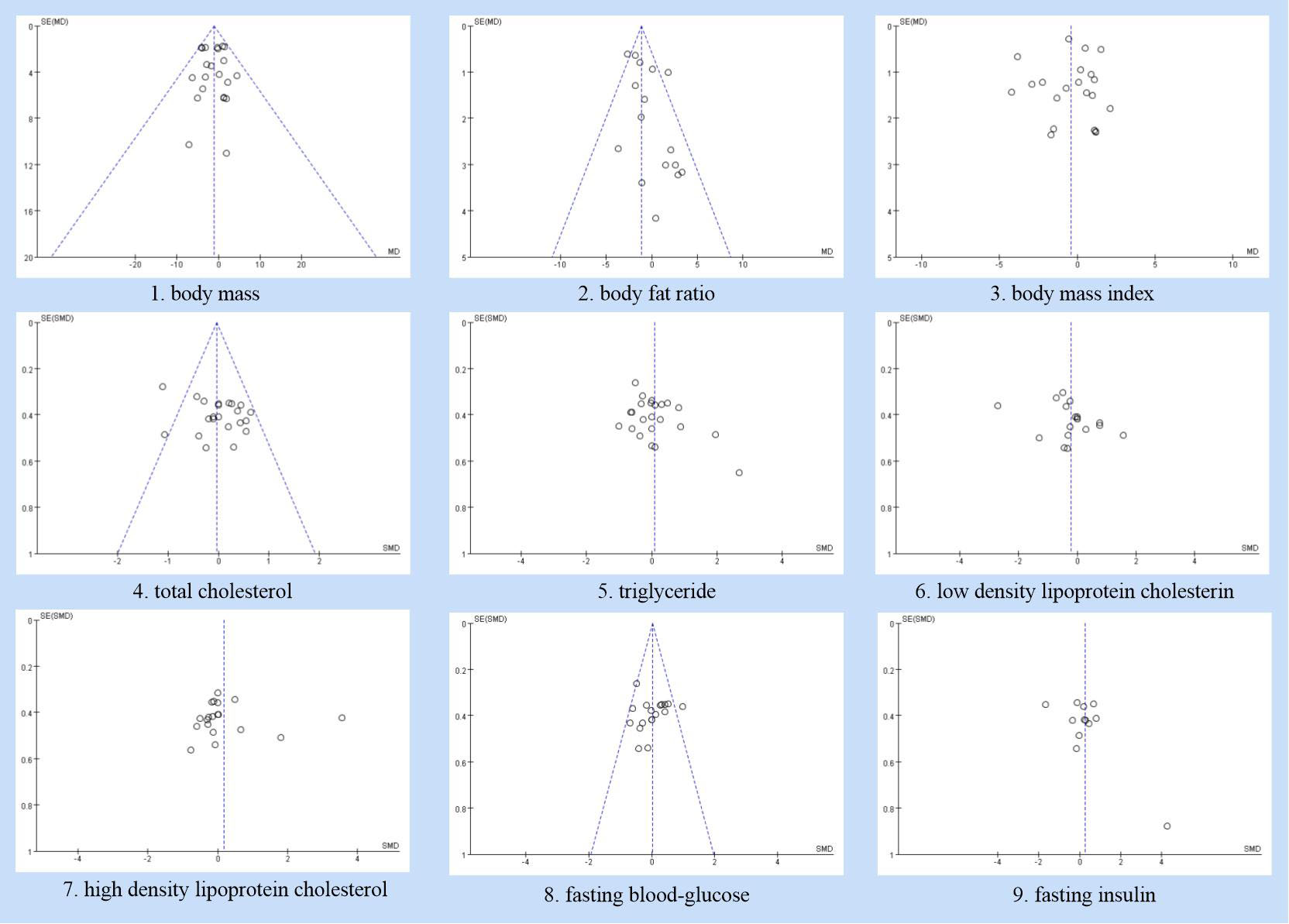

Supplement: Supplementary file 2 [file DataSheet1.zip › Supplementary Material Presentation/Figure 13.jpeg]

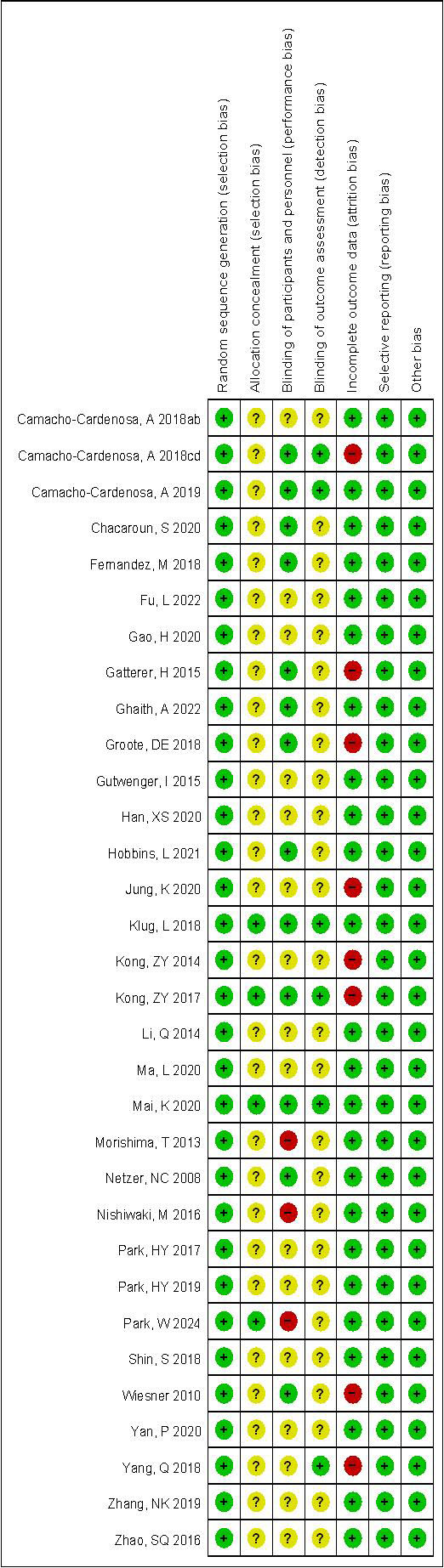

Supplement: Supplementary file 2 [file DataSheet1.zip › Supplementary Material Presentation/Figure 2.jpeg]

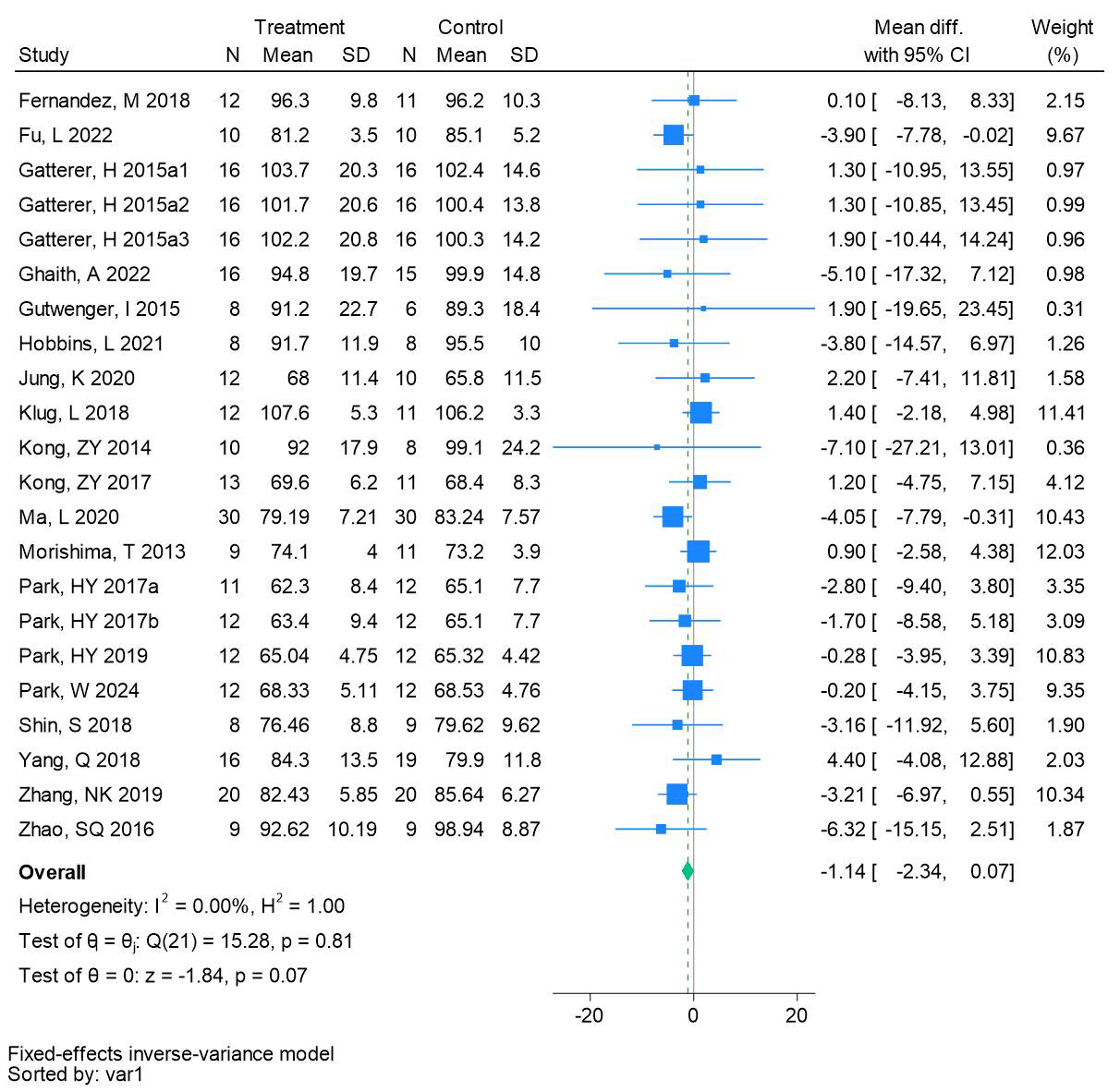

Supplement: Supplementary file 2 [file DataSheet1.zip › Supplementary Material Presentation/Figure 3.jpeg]

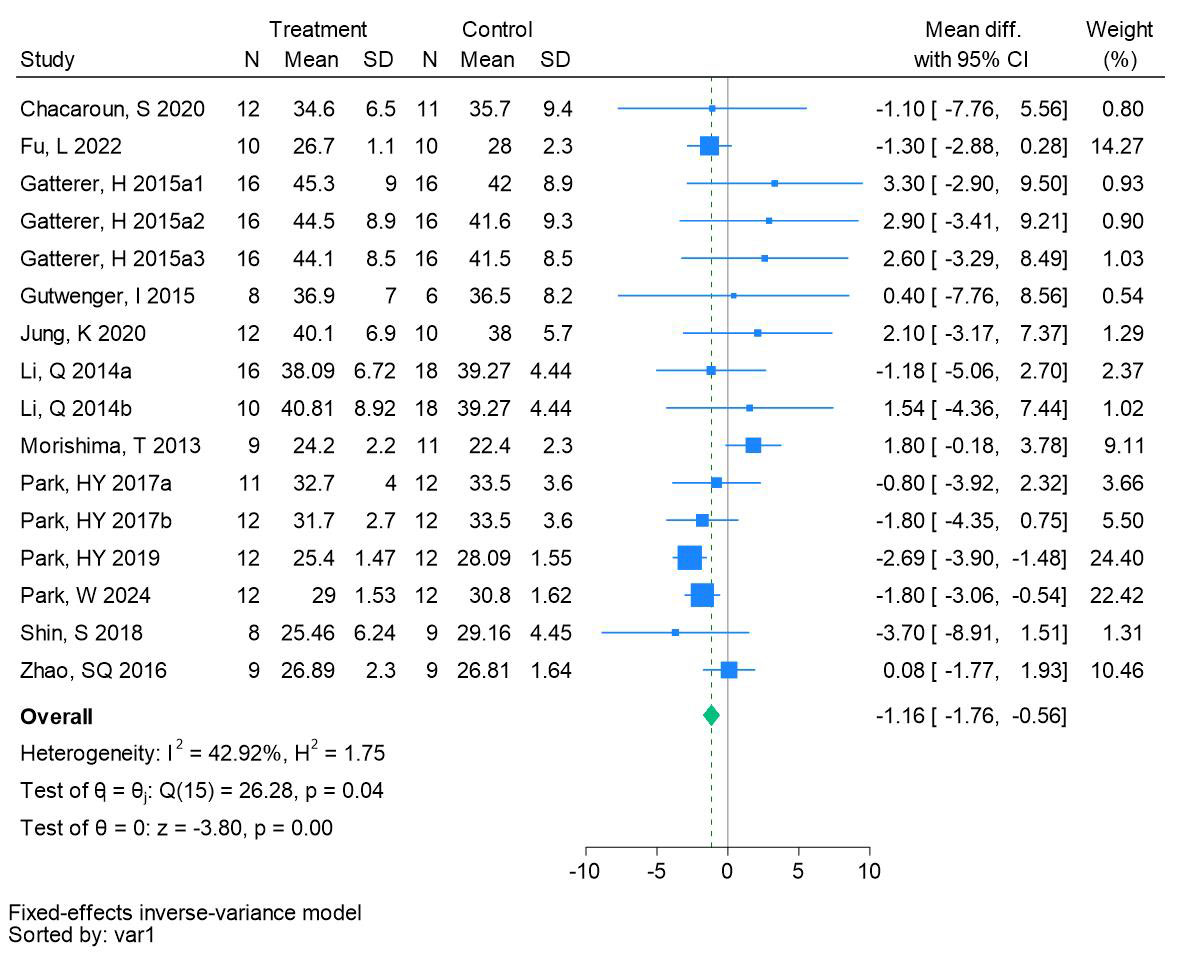

Supplement: Supplementary file 2 [file DataSheet1.zip › Supplementary Material Presentation/Figure 4.jpeg]

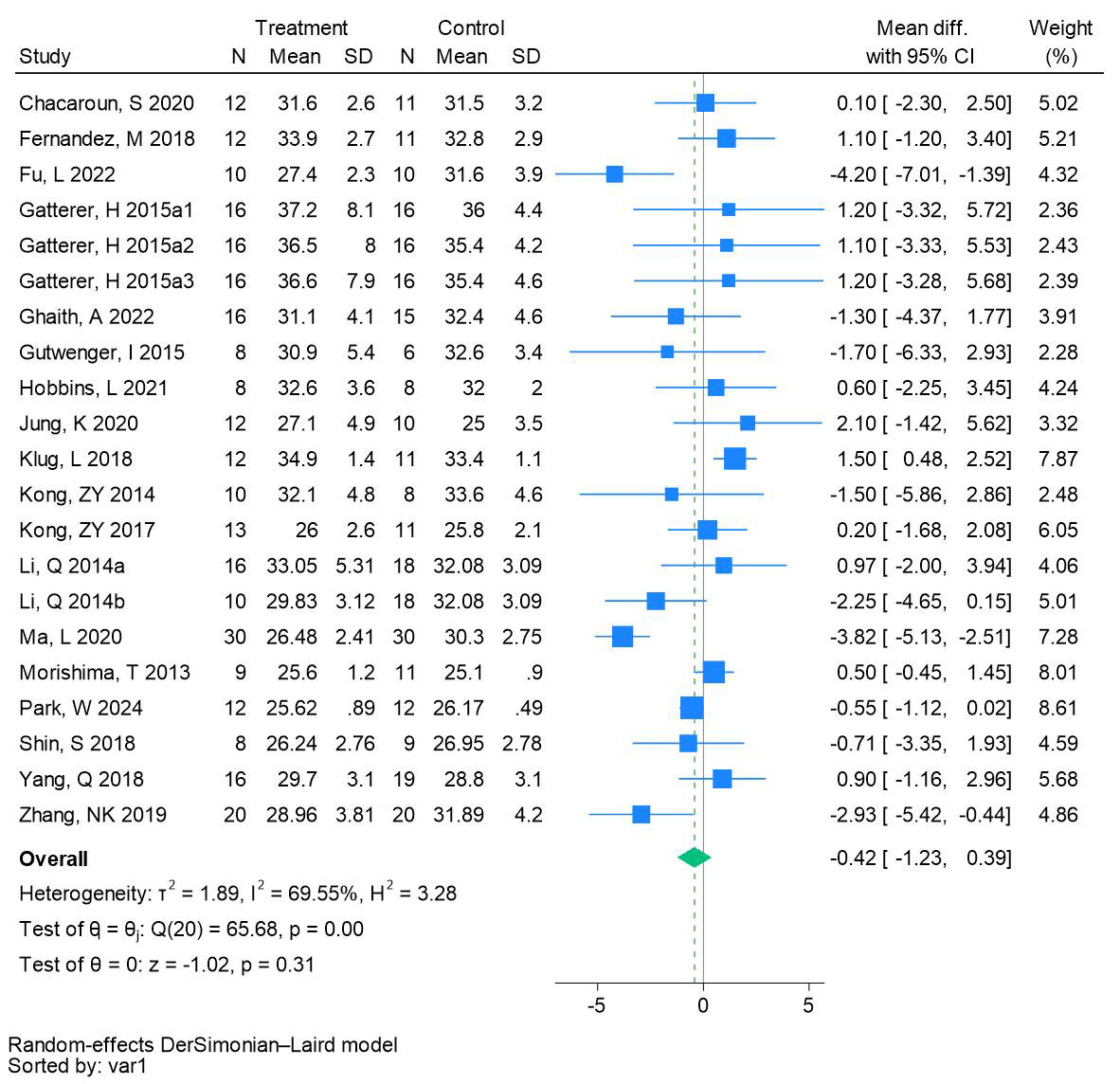

Supplement: Supplementary file 2 [file DataSheet1.zip › Supplementary Material Presentation/Figure 5.jpeg]

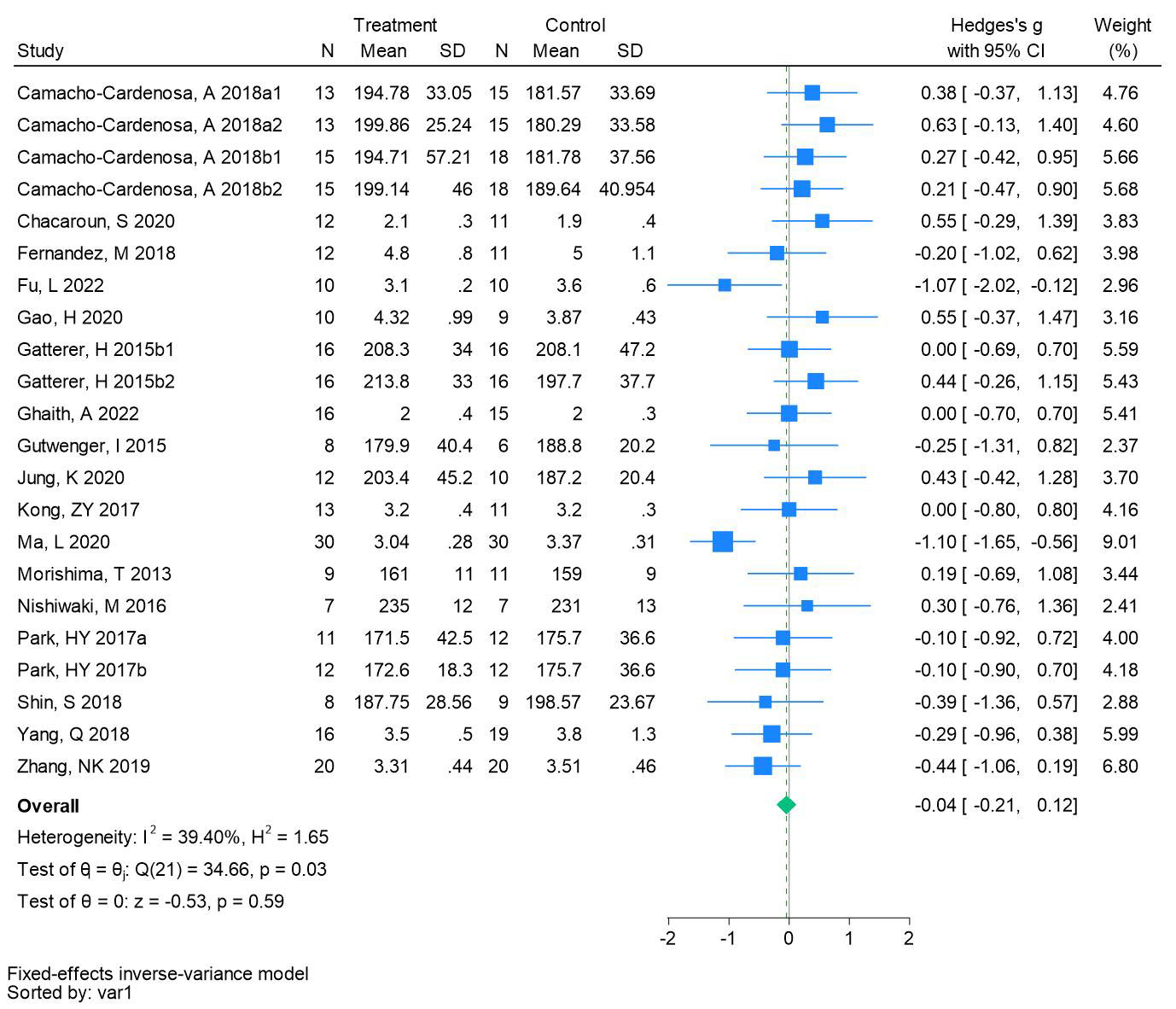

Supplement: Supplementary file 2 [file DataSheet1.zip › Supplementary Material Presentation/Figure 6.jpeg]

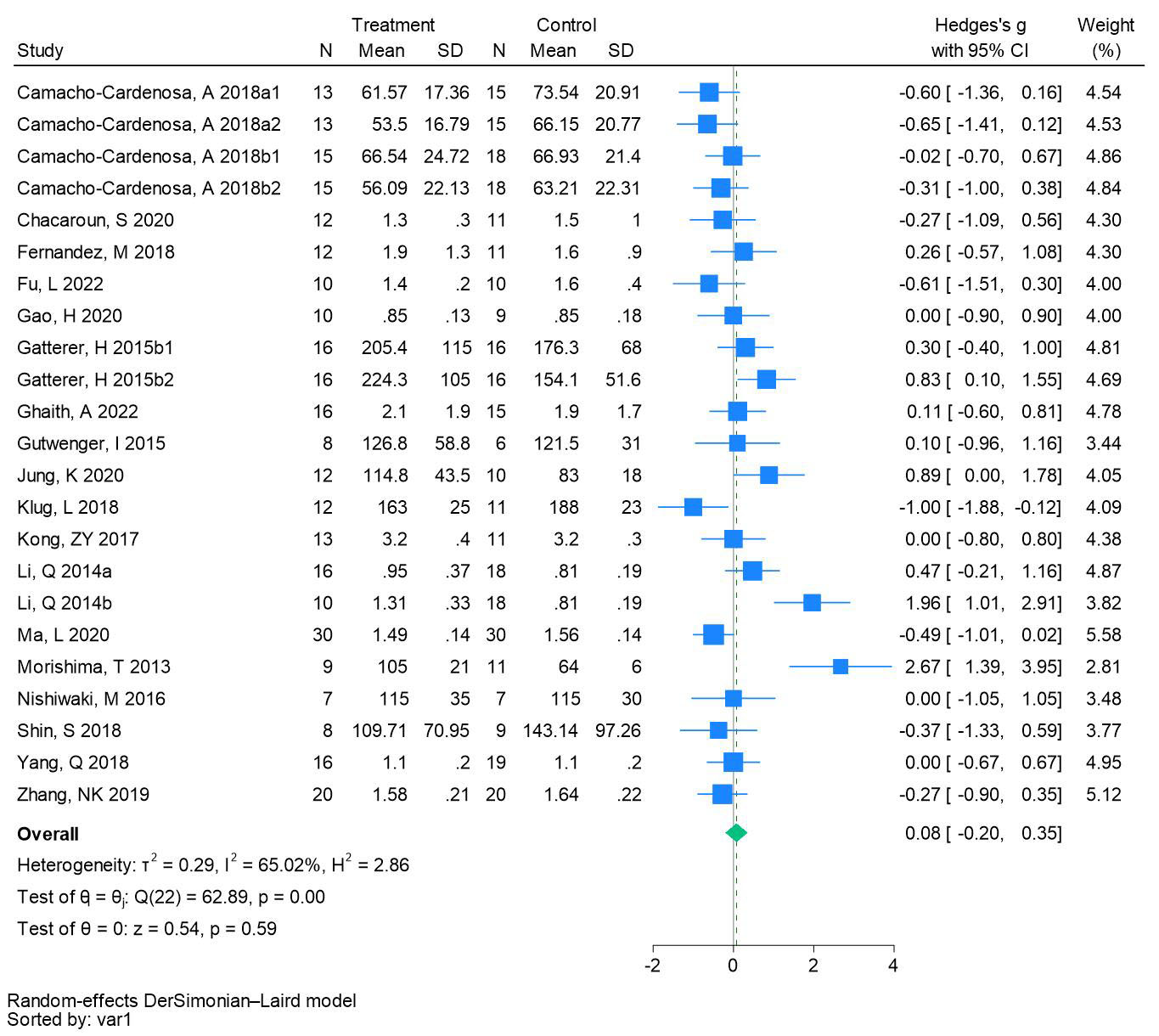

Supplement: Supplementary file 2 [file DataSheet1.zip › Supplementary Material Presentation/Figure 7.jpeg]

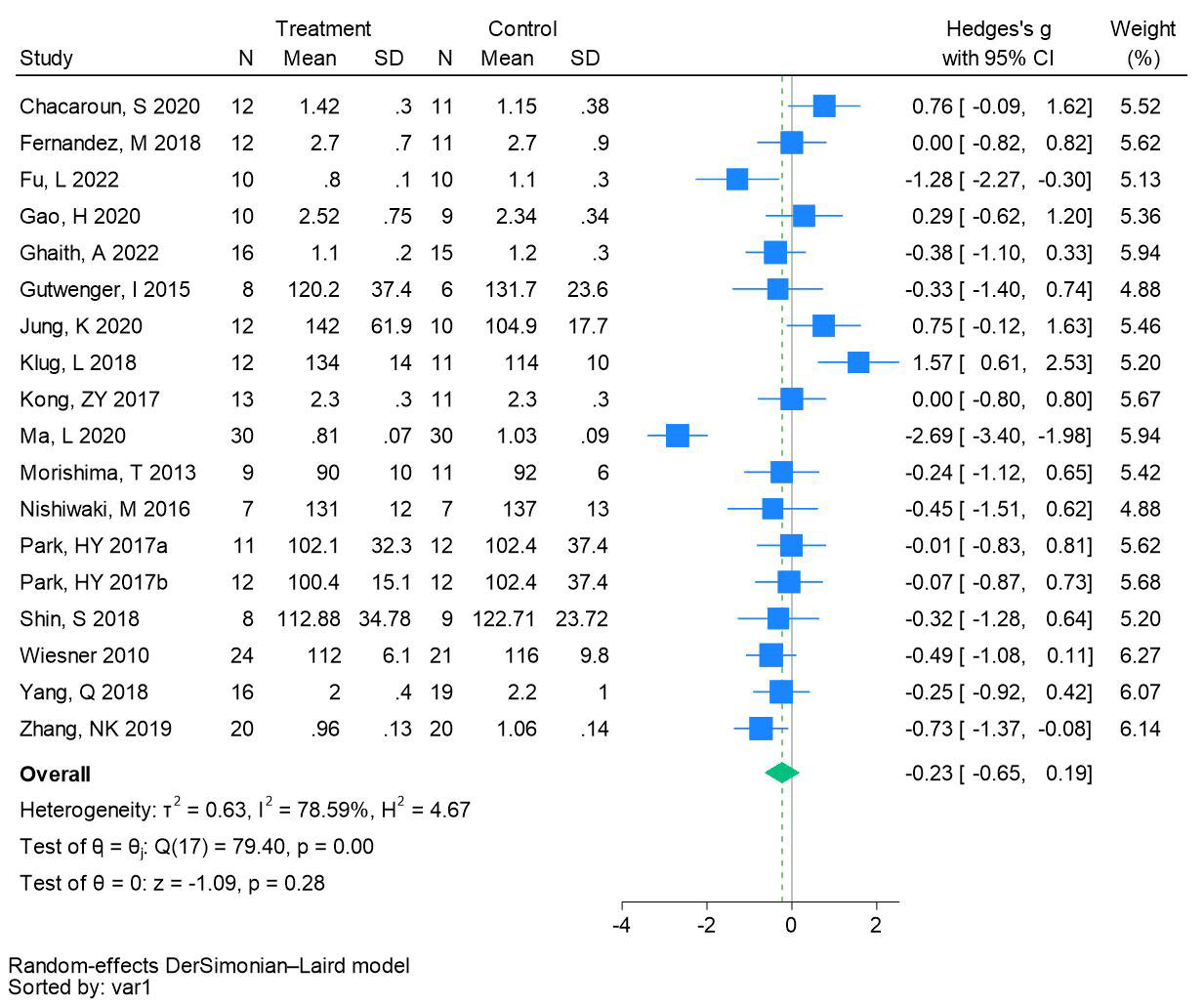

Supplement: Supplementary file 2 [file DataSheet1.zip › Supplementary Material Presentation/Figure 8.jpeg]

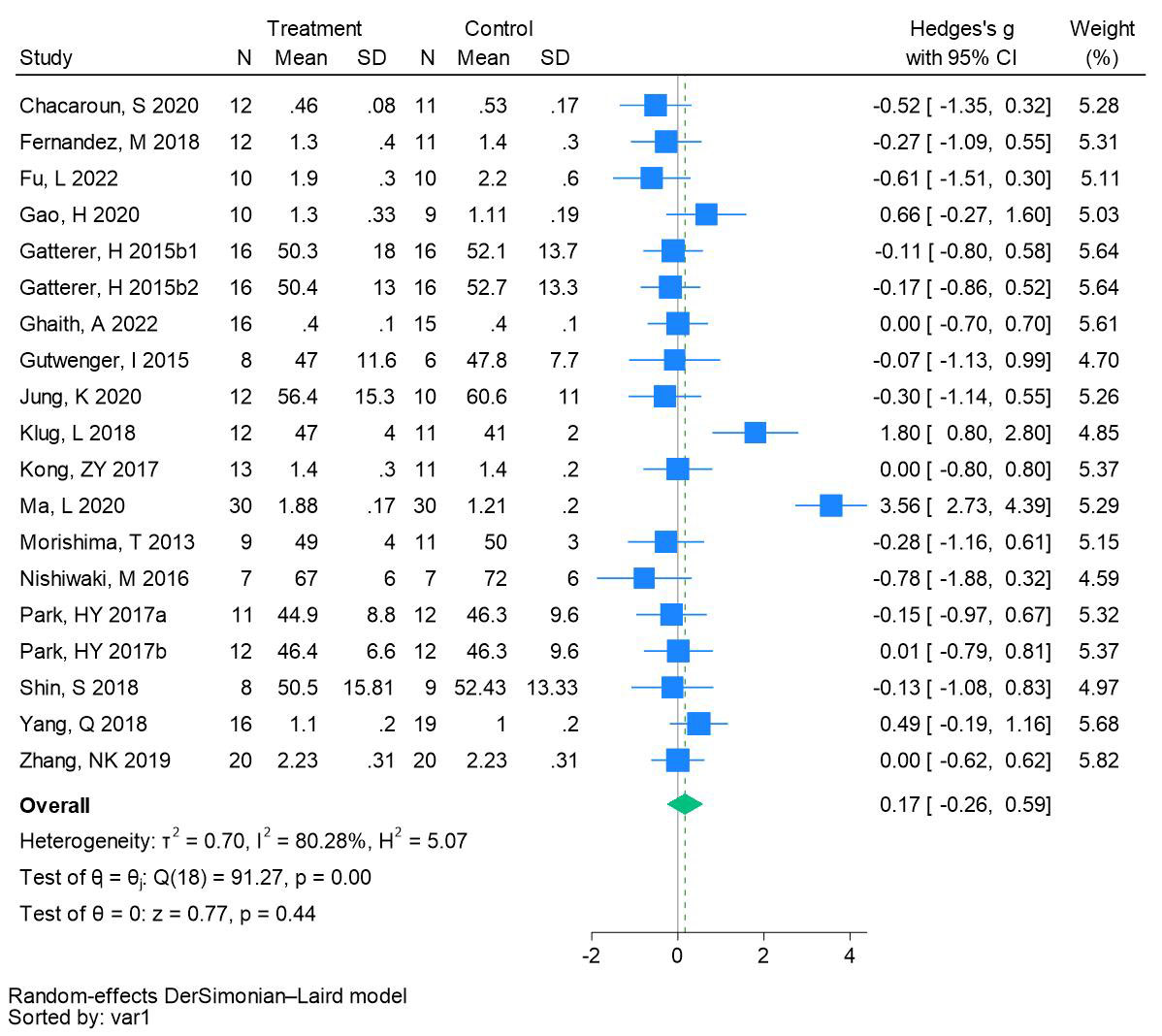

Supplement: Supplementary file 2 [file DataSheet1.zip › Supplementary Material Presentation/Figure 9.jpeg]
