# Supplementary material for: Impact of exercise training in a hypobaric/normobaric hypoxic environment on body composition and glycolipid metabolism in individuals with overweight or obesity: a systematic review and meta-analysis
Source: Front Physiol. 2025 Mar 10;16:1571730. doi: 10.3389/fphys.2025.1571730 (PMC11931047; doi:10.3389/fphys.2025.1571730)
Supplement: Supplementary file 3 [file Table2.docx]

Supplementary Table 2. Main characteristics of studies included in the meta-analysis

| Study | Basic information | | |  | Intervention program | | |  |  | Results and Comments |
| --- | --- | --- | --- | --- | --- | --- | --- | --- | --- | --- |
|  | Participants  (M/F) | Age  (M±SD)/year | BMI  (M±SD)/kg/m^2^ |  | Duration/  Frequency | Altitude/  time | Modality/  Exercise intensity |  | Outcome |  |
| Kong,  ZY  2014 | HET: 5/5  NET: 5/3 | HET:  19.8 ± 2.2  NET:  22.3 ± 1.7 | HET:  34.7 ± 5.3  NET:  33.8 ± 5.6 |  | 4 weeks  7 days/wk | 2000-  3000m  60min | Aerobic and resistance training.  60-70% HRmax |  | 1)2) | The study showed that hypoxic training was more effective in reducing body mass in obese people than normal oxygen training. However, hypoxia training has no obvious advantages over normal oxygen training in improving the BMI, fat mass, and waist-to-hip ratio of obese people. The study had a small sample size. The results were based on a combined analysis of obese men and women. |
| Yang, Q  2018 | HET: 8/8  NET: 11/8 | HET:  14.3 ± 1.4  NET:  13.9 ± 0.9 | HET:  32.9 ± 3.5  NET:  31.5 ± 3.4 |  | 4 weeks  6 days/wk | 2700m  120min | aerobic exercise.  20-40%HRR |  | 1)2)4)5)6)7)8)9)10) | The study showed that continuous low-intensity exercise in two different environments can effectively improve the body composition and glucose and lipid metabolism of obese people. In a low-oxygen environment, the decrease in body composition is obviously better than that in a normal-oxygen environment. At the level of glucose and lipid metabolism, no advantage of hypoxic training was found. |
| Gao, H  2020 | HET: 0/10  NET: 0/9 | HET:  19.30 ± 1.92  NET:  16.61 ± 1.96 | HET:  27.97 ± 3.58  NET:  28.88 ± 4.08 |  | 4 weeks  6 days/wk | 2300m  Total 5h  hypoxia 2h | aerobic exercise.  20-40%HRR |  | 4)5)6)7) | The study did not distinguish between overweight and obesity. This study shows that four weeks of hypoxic training can effectively reduce body mass, fat mass, TC, and HDL-C levels in overweight or obese people. However, the loss of weight and fat mass did not show an additional benefit compared to normal oxygen training. |
| Li, Q  2014(a/b:Hypobaric) | HETa: 8/8  HETb: 4/6  NET: 10/8 | 16-24 | ＞27 |  | 4 weeks  6 days/wk | a: 2366m  b: 2388m  Morning: 2 h afternoon: 2 h | aerobic exercise.  40% HRR |  | 2)5)8)9)10) | Compared with low-intensity training in a normal oxygen environment, low-intensity training in a low-oxygen environment can reduce the fat mass and BMI of obese people more effectively. However, it has no obvious advantages in regulating blood sugar and improving insulin resistance. The results were based on a combined analysis of obese men and women. |
| Zhao, SQ  2016 | HET: 9/0  NET: 9/0 | HET:  18.24 ± 2.23  NET:  18.08 ± 1.79 | HET:  32.9 ± 3.1  NET:  31.5 ± 2.6 |  | 8 weeks  5 days/wk | 2700m  60min | aerobic exercise.  65-75% VO_2max_ |  | 1)3) | The study showed that in the low oxygen environment, the fat loss of obese people was significantly better than that in the normal oxygen environment, and there was a significant difference. The study had a small sample size. There was no change in exercise intensity during the 8-week exercise intervention, and it was unclear to explore the optimal balance point between exercise intensity and intervention effect. |

Supplementary Table 2. (Continued) Main characteristics of studies included in the meta-analysis

| Study | Basic information | | |  | Intervention program | | |  |  | Results and Comments |
| --- | --- | --- | --- | --- | --- | --- | --- | --- | --- | --- |
|  | Participants  (M/F) | Age  (M±SD)/year | BMI  (M±SD)/kg/m^2^ |  | Duration/  Frequency | Altitude/  time | Modality/  Exercise intensity |  | Outcome | Comments on Key Strengths/Limitations |
| Zhang, NK  2019 | HET: 20/0  NET: 20/0 | HET:  22.34 ± 2.15  NET:  21.87 ± 2.37 | HET:  32.78 ± 4.31  NET:  33.32 ± 4.38 |  | 4 weeks  5 days/wk | 2500m  60min | aerobic exercise.  65-75% VO_2max_ |  | 1)2)4)5)6)7) | Compared with normal oxygen moderate intensity exercise, low oxygen moderate intensity exercise can effectively reduce the body mass, fat mass, BMI, and lipid metabolism level of obese people, and can avoid the weight rebound after exercise, so that the lipid level is maintained at a normal level. |
| Ma, L  2020 | HET: 30/0  NET: 30/0 | HET:  22.14 ± 1.66  NET:  22.08 ± 2.02 | HET:  33.42 ± 3.04  NET:  33.32 ± 4.38 |  | 4 weeks  5 days/wk | 2500m  60min | aerobic exercise.  65% VO_2max_ |  | 1)2)4)5)6)7)8) | This study shows that compared with a normal oxygen environment, moderate intensity training in a low oxygen environment can effectively reduce the level of lipid metabolism in obese people. The decrease in body mass, fat mass, and BMI was significantly better than that under a normal oxygen environment, and there was a significant difference. However, there was no statistically significant improvement in blood sugar. |
| [Park, W 2024](https://pubmed.ncbi.nlm.nih.gov/?size=200&term=Park+W&cauthor_id=39337228) | HET: 0/12  NET: 0/12 | HET:  67.83 ± 1.03  NET:  68.08 ± 0.90 | HET:  27.31 ± 0.66  NET:  26.87 ± 0.38 |  | 12 weeks  3 days/wk | 3000m  60min | resistance training.  35min  aerobic exercise.  60-70% HRmax |  | 1)2)3) | This paper provides valuable insights for managing obesity-related cardiovascular risk factors in an aging population. Studies have shown that combining exercise training with a low-oxygen environment helps improve body composition in obese older women. |
| [Park, HY 2019](https://pubmed.ncbi.nlm.nih.gov/?size=200&term=Park+HY&cauthor_id=30788892) | HET: 12/0  NET: 12/0 | HET:  66.50 ± 0.90  NET:  66.50 ± 0.67 | HET:  26.00 ± 0.61  NET:  25.63 ± 0.35 |  | 12 weeks  3 days/wk | 3000m  60min | resistance training.  30-40min  aerobic exercise.  60-70% HRmax |  | 1)3) | This paper shows that hypoxic training can effectively promote the health of obese elderly people. Compared with normal oxygen training, hypoxic training can improve the body composition of obese elderly men more effectively. |
| Fu, L  2022 | HET: 10/0  NET: 10/0 | HET:  22.3 ± 2.8  NET:  22.5 ± 2.6 | HET:  34.7 ± 4.2  NET:  34.5 ± 4.6 |  | 4 weeks  5 days/wk | 2300m  60min | aerobic exercise.  65% VO_2max_ |  | 1)2)3)4)5)6)7) | The literature shows that hypoxic training is worth popularizing and applying. It is helpful to improve the body composition and lipid metabolism level of obese poople. |

Supplementary Table 2. (Continued) Main characteristics of studies included in the meta-analysis

| Study | Basic information | | |  | Intervention program | | |  |  | Results and Comments |
| --- | --- | --- | --- | --- | --- | --- | --- | --- | --- | --- |
|  | Participants  (M/F) | Age  (M±SD)/year | BMI  (M±SD)/kg/m^2^ |  | Duration/  Frequency | Altitude/  time | Modality/  Exercise intensity |  | Outcome | Comments on Key Strengths/Limitations |
| [Chacaroun, S 2020](https://pubmed.ncbi.nlm.nih.gov/?size=200&term=Chacaroun+S&cauthor_id=32102058) | HET: 11/1  NET: 8/3 | HET:  52 ± 12  NET:  56 ± 11 | HET:  31.2 ± 2.4  NET:  31.8 ± 3.2 |  | 8 weeks  3 days/wk | 3700m  45min | aerobic exercise.  75% VO_2max_ |  | 2)3)4)5)6)7)8)9)10) | In this study, neither hypoxia nor normoxia training caused significant changes in body composition. This may be due to a lack of dietary control, and further research should combine hypoxic training with dietary interventions. |
| [Camacho-Cardenosa, A 2018a](https://pubmed.ncbi.nlm.nih.gov/?size=200&term=Camacho-Cardenosa+A&cauthor_id=30204493) | HET: 0/13  NET: 0/15 | HET:  44.43 ± 7.18  NET:  43.14 ± 7.67 | HET:  30.03 ± 6.37  NET:  29.59 ± 5.25 |  | a1: 6 weeks  3 days/wk  a2: 12 weeks  3 days/wk | 2500m  (24-36/36-42min) | High intensity interval training |  | 4)5)8) | a: 3-minute high-intensity exercise (90% Wmax) followed by 3 minutes of active recovery (55%-65% Wmax)  b: 30 seconds of all-out effort (130% Wmax) followed by 3 minutes of active recovery (55%-65% Wmax).  The study did not distinguish between overweight and obesity. Studies have shown that high-intensity training in a low-oxygen environment is more effective in improving abdominal fat in overweight or obese women. |
| [Camacho-Cardenosa, A 2018b](https://pubmed.ncbi.nlm.nih.gov/?size=200&term=Camacho-Cardenosa+A&cauthor_id=30204493) | HET: 0/15  NET: 0/18 | HET:  37.4 ± 10.25  NET:  40.05 ± 8.66 | HET:  27.71 ± 4.55  NET:  28.74 ± 4.77 |  | b1: 6 weeks  3 days/wk  b2: 12 weeks  3 days/wk | 2500m  (16-24/24-27min) | High intensity full sprint |  | 4)5)8) |  |
| [Jung, K 2020](https://pubmed.ncbi.nlm.nih.gov/?size=200&term=Jung+K&cauthor_id=33008106) | HET: 0/12  NET: 0/10 | HET:  47.2 ± 6.4  NET:  43.8 ± 8.6 | HET:  27.1 ± 4.3  NET:  25.1 ± 3.3 |  | 12 weeks  3 days/wk | 3000m  50min | Pilates training. |  | 1)2)3)4)5)6)7)8)9)10) | This study showed that pilates exercise in a hypoxic environment did not have a good effect on body composition, but could induce a decrease in TC and TG levels in obese women. The study did not investigate the participants' dietary intake and daily activities during the intervention period. |
| [Gatterer, H 2015a](https://pubmed.ncbi.nlm.nih.gov/?size=200&term=Gatterer+H&cauthor_id=26008855) | HET: 4/12  NET: 10/6 | HET:  50.3 ± 10.3  NET:  52.4 ± 7.9 | HET:  37.9 ± 8.1  NET:  36.3 ± 4.2 |  | a1: 5 weeks  2 days/wk  a2: 3 months  2 days/wk  a3: 8 months  2 days/wk | 3500m  90min | aerobic exercise.  65-70% HRmax |  | 1)2)3) | This was a long-term study, and the study did not prove that the hypoxic environment could have additional effects. In the long-term study, the diet of the subjects was not controlled, and there was a low dependency problem. |
| [Gatterer, H 2015b](https://pubmed.ncbi.nlm.nih.gov/?size=200&term=Gatterer+H&cauthor_id=26008855) | HET: 4/12  NET: 10/6 | HET:  50.3 ± 10.3  NET:  52.4 ± 7.9 | HET:  37.9 ± 8.1  NET:  36.3 ± 4.2 |  | b1: 3 months  2 days/wk  b2: 8 months  2 days/wk | 3500m  90min | aerobic exercise.  65-70% HRmax |  | 4)5)7)8) |  |

Supplementary Table 2. (Continued) Main characteristics of studies included in the meta-analysis

| Study | Basic information | | |  | Intervention program | | |  |  | Results and Comments |
| --- | --- | --- | --- | --- | --- | --- | --- | --- | --- | --- |
|  | Participants  (M/F) | Age  (M±SD)/year | BMI  (M±SD)/kg/m^2^ |  | Duration/  Frequency | Altitude/  time | Modality/  Exercise intensity |  | Outcome | Comments on Key Strengths/Limitations |
| [Hobbins, L 2021](https://pubmed.ncbi.nlm.nih.gov/?size=200&term=Hobbins+L&cauthor_id=33707985) | HET: 4/4  NET: 5/3 | HET:  32.1 ± 10.2  NET:  41.1 ± 13.0 | HET:  31.9 ± 3.6  NET:  33.0 ± 1.4 |  | 2 weeks  4 days/wk | 3500m  60min | self-paced interval-walking training.  RPE: 14 |  | 1)2) | The study had a shorter intervention period of 2 weeks and a smaller sample size. Hypoxia combined with low-intensity exercise did not have significant effects on the subjects. |
| Kong, ZY 2017 | HET: 0/11  NET: 0/13 | 18-30 | HET:  26.0 ± 2.4  NET:  25.7 ± 2.2 |  | 5 weeks  4 days/wk | 2500m  <45min | 60 repetitions of 8 s maximal cycling effort interspersed with 12-s recovery |  | 1)2)4)5)6)7) | The five-week double-blind study showed that high-intensity training in a low-oxygen environment did not have a good effect on blood lipids. |
| Fernandez, M 2018 | HET: 2/10  NET: 2/9 | HET:  34.8 ± 4.7  NET:  32.2 ± 8.4 | HET:  34.1 ± 2.6  NET:  32.9 ± 2.7 |  | 3 weeks  7 days/wk | 3000m  60min | walking training.  PWS |  | 1)2)4)5)6)7)8)9)10) | This study used walking training as an intervention, which was of low intensity and did not bring good benefits to body composition and glycolipid metabolism. |
| [Morishima, T 2013](https://pubmed.ncbi.nlm.nih.gov/?size=200&term=Morishima+T&cauthor_id=23879294) | HET: 9/0  NET: 11/0 | HET:  30 ±  2  NET:  32 ± 3 | HET:  25.6 ± 1.2  NET:  25.4 ± 0.9 |  | 4 weeks  3 days/wk | 2700m  60min | aerobic exercise.  55% VO_2max_ |  | 1)2)3)4)5)6)7)8)9) | The study showed that 4 weeks of hypoxic training resulted in greater improvements in glucose tolerance, with no significant benefits for improvements in body composition. |
| Nishiwaki, M 2016 | HET: 0/7  NET: 0/7 | 56 ± 1 | HET:  24.6 ± 0.8  NET:  ＞24 |  | 8 weeks  4 days/wk | 2000m  30min | underwater training.  50% VO_2max_ |  | 4)5)6)7)8) | This study shows that exercise training under mildly hypoxic conditions can more effectively reduce body mass, BMI and body fat percentage in postmenopausal women in a shorter period of time. |
| Wiesner 2010 | HET: 10/14  NET: 8/13 | HET:  42.2 ± 1. 2  NET:  42.1 ± 1.7 | HET:  33.1 ± 0.3  NET:  32.5 ± 0.8 |  | 4 weeks  3 days/wk | 2740m  60min | aerobic exercise.  65% VO_2max_ |  | 6)9)10) | Hypoxic training had a greater improvement in body composition, but did not have a better effect on glucose metabolism. |

Supplementary Table 2. (Continued) Main characteristics of studies included in the meta-analysis

| Study | Basic information | | |  | Intervention program | | |  |  | Results and Comments |
| --- | --- | --- | --- | --- | --- | --- | --- | --- | --- | --- |
|  | Participants  (M/F) | Age  (M±SD)/year | BMI  (M±SD)/kg/m^2^ |  | Duration/  Frequency | Altitude | Modality/  Exercise intensity |  | Outcome | Comments on Key Strengths/Limitations |
| [Park, HY 2017](https://www.researchgate.net/profile/Hun-Young-Park?_tp=eyJjb250ZXh0Ijp7ImZpcnN0UGFnZSI6InB1YmxpY2F0aW9uIiwicGFnZSI6InB1YmxpY2F0aW9uIn19)(a/b) | HETa: 0/11  HETb: 0/12  NET: 0/12 | HETa:  42.0 ± 4.4  HETb:  46.6 ± 5.7  NET:  47.2 ± 6.3 | ＞30 |  | 6 weeks  5 days/wk | a: 2000m  b: 3000m  60min | aerobic exercise.  75% HRmax |  | 1)3)4)6)7) | Hypoxic training has a better effect on the reduction of body fat and body fat percentage. Hypoxic training resulted in greater reductions in total cholesterol and LDL cholesterol. |
| [Shin, S 2018](https://scholar.cnki.net/home/search?sw=6&sw-input=Sohee Shin" \o "https://scholar.cnki.net/home/search?sw=6&sw-input=Sohee Shin) | HET: 8/0  NET: 9/0 | HET:  45.6 ± 20.9  NET:  46.0 ± 20.5 | HET:  26.8 ± 2.3  NET:  27.0 ± 3.0 |  | 4 weeks  3 days/wk | 2500m  50min | aerobic exercise.  60% HRmax |  | 1)2)3)4)5)6)7)9)10) | This is a study of people with metabolic syndrome. Hypoxic training improved body weight, body fat percentage, BMI, TC and LDL-C. |
| [Gutwenger, I 2015](https://pubmed.ncbi.nlm.nih.gov/?term=[Author]" \o "https://pubmed.ncbi.nlm.nih.gov/?term=[Author])  hypobaric | HET: 3/5  NET: 3/3 | HET:  50.1 ± 7.8  NET:  63.3 ± 5.2 | HET:  31.1 ± 5.3  NET:  32.3 ± 4.2 |  | 2 weeks  4 days/wk | 1900m  180min | hiking  55-65% HRmax |  | 1)2)3)4)5)6)7)8)9) | This is a study of subjects with metabolic syndrome that was conducted for only 2 weeks without dietary control. The results showed that hypoxia training could improve TG concentration. |
| [Ghaith, A 2022](https://pubmed.ncbi.nlm.nih.gov/?size=200&term=Ghaith+A&cauthor_id=36121143) | HET: 10/6  NET: 13/2 | HET:  51.0 ± 8.3  NET:  52.0 ± 7.5 | HET:  31.5 ± 4.0  NET:  32.4 ± 4.8 |  | 8 weeks  3 days/wk | 4200m  60min | cycling at 80% or 100% of maximal workload |  | 1)2)4)5)6)7)8)9)10) | The combination of anoxic environment and high intensity did not produce better benefits. There is no guarantee that subjects will maintain the same calorie intake and diet during the exercise program. |
| [Klug, L 2018](https://pubmed.ncbi.nlm.nih.gov/?size=200&term=Klug+L&cauthor_id=30565412) | HET: 12/0  NET: 11/0 | HET:  55.0 ± 2.1  NET:  57.6 ± 2.2 | HET:  35.5 ± 1.4  NET:  34.1 ± 0.9 |  | 6 weeks  3 days/wk | 2500m  60min | aerobic exercise.  50-60% HRmax |  | 1)2)3)5)6)7)8)9) | This is a study of people with metabolic syndrome. The lower intensity of exercise may be the reason why their body composition and sugar metabolism did not send significant changes. |
| [Mai](https://pubmed.ncbi.nlm.nih.gov/?size=200&term=Mai+K&cauthor_id=31819201). K  2020 | HET: 12/0  NET: 11/0 | HET:  57  NET:  58 | HET:  33.5  NET:  34.2 |  | 6 weeks  3 days/wk | 2500m  60min | interval training.  60% VO_2max_ |  | 2)10) | This is a study of people with metabolic syndrome. This study suggests that physical exercise under hypoxic conditions can partially enhance the insulin sensitivity of muscle cells in obese men with metabolic syndrome who exercise alone. |

Supplementary Table 2. (Continued) Main characteristics of studies included in the meta-analysis

| Study | Basic information | | |  | Intervention program | | |  |  | Results and Comments |
| --- | --- | --- | --- | --- | --- | --- | --- | --- | --- | --- |
|  | Participants  (M/F) | Age  (M±SD)/year | BMI  (M±SD)/kg/m^2^ |  | Duration/  Frequency | Altitude | Modality/  Exercise intensity |  | Outcome | Comments on Key Strengths/Limitations |
| [Camacho-Cardenosa, A 2019a](https://pubmed.ncbi.nlm.nih.gov/?size=200&term=Camacho-Cardenosa+A&cauthor_id=30204493) | HET: 0/13  NET: 0/15 | 40.6 ± 9.5 | 28.00 ± 5.32 |  | 12 weeks  3 days/wk | 2500m  41.5min | High intensity interval training |  | 4)5)8) | a: performed 3 minutes of high‐intensity exercise (90% Wmax followed by 3 minutes of active recovery [55%‐65% Wmax]).  b: underwent 30 seconds of all‐out (130% Wmax) followed by 3 minutes of active recovery at 55%‐65% Wmax.  The study did not distinguish between overweight and obesity. High-intensity training under hypoxia conditions showed significant positive effects on waist circumference, WHR and percentage of trunk fat mass. The study looked at stopping training for four weeks after 12 weeks of training and found that the percentage of body fat mass still decreased significantly. |
| [Camacho-Cardenosa, A 2019b](https://pubmed.ncbi.nlm.nih.gov/?size=200&term=Camacho-Cardenosa+A&cauthor_id=30204493) | HET: 0/15  NET: 0/18 | 40.6 ± 9.5 | 28.00 ± 5.32 |  | 12 weeks  3 days/wk | 2500m  41.5min | High intensity full sprint |  | 4)5)8) |  |
| Yan, P  2020 | HET: 30/0  NET: 30/0 | HET:  22.45 ± 2.32  NET:  22.11 ± 2.45 | HET:  30.63 ± 1.63  NET:  30.18 ± 1.24 |  | 4 weeks  5 days/wk | 2500m  60min | aerobic exercise.  65% VO_2max_ |  | 1)2)4)5)6)7) | Hypoxic moderate intensity training can significantly reduce body composition and improve lipid metabolism in obese patients. |
| Han, XS  2020 | HET: 20/0  NET: 20/0 | HET:  22.32 ± 1.27  NET:  21.54 ± 2.21 | HET:  30.49 ± 1.09  NET:  30.79 ± 1.36 |  | 8 weeks  4 days/wk | 3000m  60min | aerobic exercise.  70% VO_2max_ |  | 2)4)5)6)7) | Hypoxic medium intensity exercise has a good effect on lipid reduction, has a good intervention effect on lipid metabolism, and can effectively improve exercise ability. |
| [Netzer](https://pubmed.ncbi.nlm.nih.gov/?term="Netzer NC"[Author]), N  2008 | HET: 2/8  NET: 2/8 | HET:  50.1  NET:  45.5 | HET:  33.4  NET:  32.8 |  | 8 weeks  3 days/wk | 2500m  90min | aerobic exercise.  60% VO_2max_ |  | 1)2)4)5)6)7) | This is the first study of hypoxic training on obesity, which shows that hypoxic training produces better benefits for body mass and BMI. |
| [Groote](https://pubmed.ncbi.nlm.nih.gov/?size=200&term=DE+Groote+E&cauthor_id=29923910), ED  2018 | HET: 3/4  NET: 3/4 | 12-15 | ＞30 |  | 6 weeks  3 days/wk | 2800m  50-60  min | combined exercise.  aerobic training combined with strength training. |  | 1)2)3)4)5)8)9)10) | This is a combination of training, and studies have shown that hypoxia is particularly effective in terms of glucose tolerance and insulin's response to glucose challenges. The researchers were young and the data collection did not use the most accurate instruments. |

Supplementary Table 2. (Continued) Main characteristics of studies included in the meta-analysis

| Study | Basic information | | |  | Intervention program | | |  |  | Results and Comments |
| --- | --- | --- | --- | --- | --- | --- | --- | --- | --- | --- |
|  | Participants  (M/F) | Age  (M±SD)/year | BMI  (M±SD)/kg/m^2^ |  | Duration/  Frequency | Altitude | Modality/  Exercise intensity |  | Outcome | Comments on Key Strengths/Limitations |
| [Camacho-Cardenosa, A 2018c](https://pubmed.ncbi.nlm.nih.gov/?size=200&term=Camacho-Cardenosa+A&cauthor_id=30204493) | HET: 0/13  NET: 0/13 | HET:  44.43 ± 7.18  NET:  43.14 ± 7.67 | HET:  30.03 ± 6.37  NET:  29.59 ± 5.25 |  | 12 weeks  3 days/wk | 2500m  41.5min | High intensity interval training |  | 1)2)3) | c: performed 3 minutes of high‐intensity exercise (90% Wmax followed by 3 minutes of active recovery [55%‐65% Wmax]).  d: underwent 30 seconds of all‐out (130% Wmax) followed by 3 minutes of active recovery at 55%‐65% Wmax.  The study did not distinguish between overweight and obesity. The results showed that HIIT after 12 weeks of intermittent hypoxia at normal pressure was expected to reduce body fat content and increase muscle mass. |
| [Camacho-Cardenosa, A 2018d](https://pubmed.ncbi.nlm.nih.gov/?size=200&term=Camacho-Cardenosa+A&cauthor_id=30204493) | HET: 0/18  NET: 0/15 | HET:  37.40± 10.25  NET:  40.05 ± 8.66 | HET:  27.71 ± 4.55  NET:  28.74 ± 4.77 |  | 12 weeks  3 days/wk | 2500m  41.5min | High intensity interval training |  | 1)2)3) |  |

Note: M, male; F, female; ; wk, weeks; HRmax, max heart rate; HRR, heart rate reserve; VO_2max_, maximal oxygen consumption; 1):Body mass; 2):Body mass index; 3): Body fat ratio; 4):Total cholesterol; 5):Triglycerides; 6):Low-density lipoprotein cholesterol; 7):High-density lipoprotein cholesterol ; 8):Fasting blood glucose; 9):Fasting blood insulin; 10):homeostatic assessment of insulin resistance.
