# Supplementary material for: Impact of exercise training in a hypobaric/normobaric hypoxic environment on body composition and glycolipid metabolism in individuals with overweight or obesity: a systematic review and meta-analysis
Source: Front Physiol. 2025 Mar 10;16:1571730. doi: 10.3389/fphys.2025.1571730 (PMC11931047; doi:10.3389/fphys.2025.1571730)
Supplement: Supplementary file 4 [file Table3.docx]

Supplementary Material

Supplementary Table 3. Certainty of evidence for meta-analysed outcomes

| Outcome | No of Participants and Studies | Gradeassessment | | | | | Certainty of evidence |
| --- | --- | --- | --- | --- | --- | --- | --- |
|  |  | Risk of bias | Inconsistency | Indirectness | Imprecision | Publication bias |  |
| BM | 572 (22 RCTs) | Serious^a^ | Not serious | Not serious | Serious^c^ | Not serious | Low |
| BFR | 387 (16 RCTs) | Serious^a^ | Not serious | Not serious | Serious^cd^ | Not serious | Low |
| BMI | 568 (21 RCTs) | Serious^a^ | Serious^b^ | Not serious | Serious^c^ | Not serious | Very low |
| TC | 595 (22 RCTs) | Serious^a^ | Not serious | Not serious | Serious^c^ | Not serious | Low |
| TG | 633 (23 RCTs) | Serious^a^ | Serious^b^ | Not serious | Serious^c^ | Not serious | Very low |
| LDL - C | 477 (18 RCTs) | Serious^a^ | Serious^b^ | Not serious | Serious^c^ | Not serious | Very low |
| HDL - C | 496 (19 RCTs) | Serious^a^ | Serious^b^ | Not serious | Serious^c^ | Not serious | Very low |
| FBG | 513 (18 RCTs) | Serious^a^ | Not serious | Not serious | Serious^c^ | Not serious | Low |
| BFI | 315 (12 RCTs) | Serious^a^ | Serious^b^ | Not serious | Serious^cd^ | Not serious | Very low |
| HOMA - IR | 258 (9 RCTs) | Serious^a^ | Serious^b^ | Not serious | Serious^cd^ | Not serious | Very low |

a: High risk of bias with allocation concealment.

b: High heterogeneity.

c: The confidence intervals indicated the potential for import harm or benefit.

d: Sample size not more than 400.
