# Supplementary material for: Impact of exercise training in a hypobaric/normobaric hypoxic environment on body composition and glycolipid metabolism in individuals with overweight or obesity: a systematic review and meta-analysis
Source: Front Physiol. 2025 Mar 10;16:1571730. doi: 10.3389/fphys.2025.1571730 (PMC11931047; doi:10.3389/fphys.2025.1571730)
Supplement: Supplementary file 5 [file Table4.docx]

Supplementary Material

Supplementary Table 4. Sensitivity analysis result

| Outcome | eliminate | n | MD/SMD(95%CI) | *I*^2^ | *P* |
| --- | --- | --- | --- | --- | --- |
| BMI | Ma, L 2020 | 20 | -0.06, -0.43 to 0.31 | 48 | 0.74 |
| TG | [Morishima, T 2013](https://pubmed.ncbi.nlm.nih.gov/?size=200&term=Morishima+T&cauthor_id=23879294) | 21 | -0.09, -0.25 to 0.08 | 30 | 0.30 |
|  | Li, Q 2014b |  |  |  |  |
| LDL - C | Ma, L 2020 | 16 | -0.20, -0.40 to 0.00 | 26 | 0.06 |
|  | [Klug, L 2018](https://pubmed.ncbi.nlm.nih.gov/?size=200&term=Klug+L&cauthor_id=30565412) |  |  |  |  |
| HDL - C | Ma, L 2020 | 18 | -0.02, -0.21 to 0.17 | 27 | 0.81 |
| FBI | [Morishima, T 2013](https://pubmed.ncbi.nlm.nih.gov/?size=200&term=Morishima+T&cauthor_id=23879294) | 10 | 0.22, -0.03 to 0.47 | 0 | 0.09 |
|  | Wiesner 2010 |  |  |  |  |
| HOMA - IR | Wiesner 2010 | 8 | 0.26, -0.02 to 0.53 | 0 | 0.07 |
